# Metabolic silencing induced by the small bacterial membrane protein YohP

**DOI:** 10.1016/j.isci.2025.114123

**Published:** 2025-11-19

**Authors:** Ana Natriashvili, Nahid Mohammadsadeghi, Martin Milanov, Eva Smudde, Isabel Prucker, Henning J. Jessen, Iulia Carabadjac, Heiko Heerklotz, Pedro H.C. Franco, Julian D. Langer, Hans-Georg Koch

**Affiliations:** 1 Institute of Biochemistry and Molecular Biology, ZBMZ, Faculty of Medicine, Albert-Ludwigs-University Freiburg, 79104 Freiburg, Germany; 2 Faculty of Biology, Albert-Ludwigs-University Freiburg, 79104 Freiburg, Germany; 3 Spemann Graduate School of Biology and Medicine, Albert-Ludwigs-University Freiburg, 79104 Freiburg, Germany; 4 Institute for Organic Chemistry, Faculty of Chemistry and Pharmacy, Albert-Ludwigs-University Freiburg, 79104 Freiburg, Germany; 5 CIBSS Centre for Integrative Biological Signalling, Albert-Ludwigs University Freiburg, 79104 Freiburg, Germany; 6 Institute of Pharmaceutical Sciences, Faculty of Chemistry and Pharmacy, Albert-Ludwigs-University Freiburg, 79104 Freiburg, Germany; 7 Leslie Dan Faculty of Pharmacy, University of Toronto, Toronto, ON M5S 3M2, Canada; 8 Mass Spectrometry and Proteomics, Max-Planck Institute for Biophysics, 60438 Frankfurt am Main, Germany

**Keywords:** Membrane architecture, Microbiology, Cell biology

## Abstract

Small membrane proteins represent an abundant and ubiquitous class of proteins that are often up-regulated when cells encounter unfavorable conditions, yet their function remains poorly understood. In bacteria, these proteins consist of typically less than 50 amino acids, without detectable catalytic activity. Thus, the benefit of producing these proteins during stress conditions is unknown. In the current study we used a multidisciplinary approach to determine the function of the 27-amino-acid-long protein YohP in *Escherichia coli*. Proteomics and lipidomics revealed that YohP production triggers the up-regulation of membrane protective proteins, increases the cardiolipin content, and leads to reduced membrane fluidity and a reduced membrane potential. Simultaneously, the stringent response is induced and many key metabolic enzymes are down-regulated. Overall, this multidisciplinary approach indicates that YohP-induced proteome and membrane changes initiate a state of metabolic silencing that protects *E. coli* against stress conditions and helps to conserve cellular resources.

## Highlights

• YohP production is up-regulated upon stress conditions and starvation
• YohP dissipates the membrane potential and induces the phage shock response
• YohP expression reduces nucleotide biosynthesis via the stringent response
• YohP-induced metabolic silencing increases the bacterial stress resistance

## Graphical abstract

**Figure:**
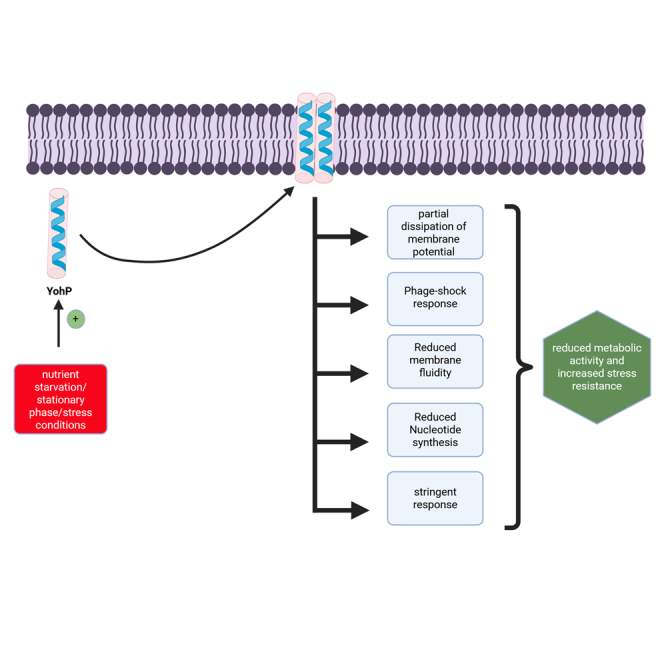

## Introduction

Bacteria are facing constant changes in their environment, and the ability to execute counteracting strategies is therefore of utmost importance for their survival. This multilayered response includes the well-established transcriptional regulation via transcription factors and regulatory RNAs,^1^^,^^2^^,^^3^^,^^4^ translational control,^5^^,^^6^^,^^7^ and the production of small stress-responsive signaling molecules, such as the hyperphosphorylated guanine nucleotides pppGpp and ppGpp.^8^^,^^9^ The synthesis of small proteins of typically less than 50 amino acids serves as an additional bacterial stress-response strategy, and this applies in particular to small membrane proteins (SMPs). These proteins consist of a single transmembrane domain with only a few amino acids residing in the cytoplasm or periplasm.^10^^,^^11^^,^^12^^,^^13^^,^^14^ Due to their small size and their predicted simple topology, SMPs are unlikely to act catalytically, but instead often execute their function by interacting with other membrane proteins. This has been shown for the SMPs MgtS and MgtU, which protect the magnesium transporters MgtA and MgtB from degradation.^15^ Another example is AcrZ, which stimulates export of antibiotics via the multi-drug efflux pump AcrAB^16^ or MgrB, which inhibits the sensor kinase PhoQ and prevents PhoQP hyperactivation.^17^ SMPs also play a crucial role in maintaining the energy balance of bacterial cells by stabilizing terminal oxidases^18^^,^^19^^,^^20^^,^^21^^,^^22^^,^^23^^,^^24^ and may facilitate the development of antimicrobial peptides.^25^

In addition to these few well-characterized examples, several hundreds of uncharacterized soluble and membrane-bound small proteins are expected to exist in *Escherichia coli*,^13^^,^^14^ for which a functional characterization is still missing. This also still applies to many of the experimentally verified small proteins.^26^^,^^27^ Examples are YncL, which consists of just 31 amino acids and which is up-regulated during heat shock in *E. coli*, or YohP, which consists of 27 amino acids and was shown to be induced in cells exposed to SDS+EDTA treatment.^28^ YohP also serves as a model protein for studying the membrane insertion pathway of SMPs. This pathway involves a post-translational recognition by the signal recognition particle (SRP) and a subsequent targeting to the SecYEG translocon or, alternatively, to the YidC insertase.^29^^,^^30^ Under stress conditions, e.g., when the SRP pathway is inhibited by accumulating (p)ppGpp,^31^ YohP insertion occurs SRP-independently via mRNA targeting.^32^

YohP is localized to the inner membrane with a mainly N_out_-C_in_ topology,^30^ but it might also acquire a dual topology, according to GFP- and PhoA-reporter fusion studies.^10^ YohP shows a strong propensity for dimerization via an unusual glycine motif,^30^ and high YohP levels cause nucleoid condensation.^30^ Nucleoid condensation via nucleoid-associated proteins is an important factor in global gene regulation in response to environmental changes. DNA-interacting proteins have only restricted access to a condensed nucleoid, which reduces gene expression and subsequently leads to decreased metabolic activity, which in turn can protect cells against unfavorable conditions.^33^ This is in line with data showing that nucleoid condensation is also observed upon induction of some membrane-permeabilizing toxins or upon contact with antimicrobial peptides.^34^^,^^35^^,^^36^^,^^37^ SMPs share some common features with membrane-acting toxins of toxin-antitoxin systems or membrane-targeting antimicrobial peptides, although they do not show strong sequence conservation on the amino acid level.^34^^,^^38^^,^^39^^,^^40^^,^^41^ Besides their small size of usually less than 50 amino acids, they all consist of a single α-helical transmembrane domain with the C terminus predicted to reside mainly in the cytosol. In addition, their transmembrane domains are often flanked by positively charged residues, which are important for membrane interaction. A typical outcome of the interaction between toxins (e.g., TisB) or antimicrobial peptides (e.g., magainin-2) and cellular membranes is the dissipation of the membrane potential.^37^^,^^40^^,^^42^^,^^43^^,^^44^ This raises the possibility that some SMPs act like antimicrobial peptides or membrane-acting toxins by partially disrupting the membrane integrity.

In the current study, we determined the consequences of YohP production in *E. coli* by complementary proteomic, lipidomic, and biochemical approaches, and our data demonstrate that YohP causes a non-lethal reduction in membrane potential, which induces a significant proteome rearrangement and stimulates the stringent response.

## Results

### YohP production is linked to nutrient starvation and stationary phase

In the *E. coli* genome, *yohP* is located between the *mdtQ* pseudogene and the gene for the tRNA-dihydrouridine^16^ synthase DusC (Figure S1). This genomic organization and the predicted amino acid sequence are conserved in most γ-proteobacteria; however, YohP homologs also exist outside of the γ-proteobacterial taxonomic group (Figure S1). The possible evolutionary relationship between different YohP homologs is also visible in a neighbor-joining tree (Figure S1).

**Figure 1:**
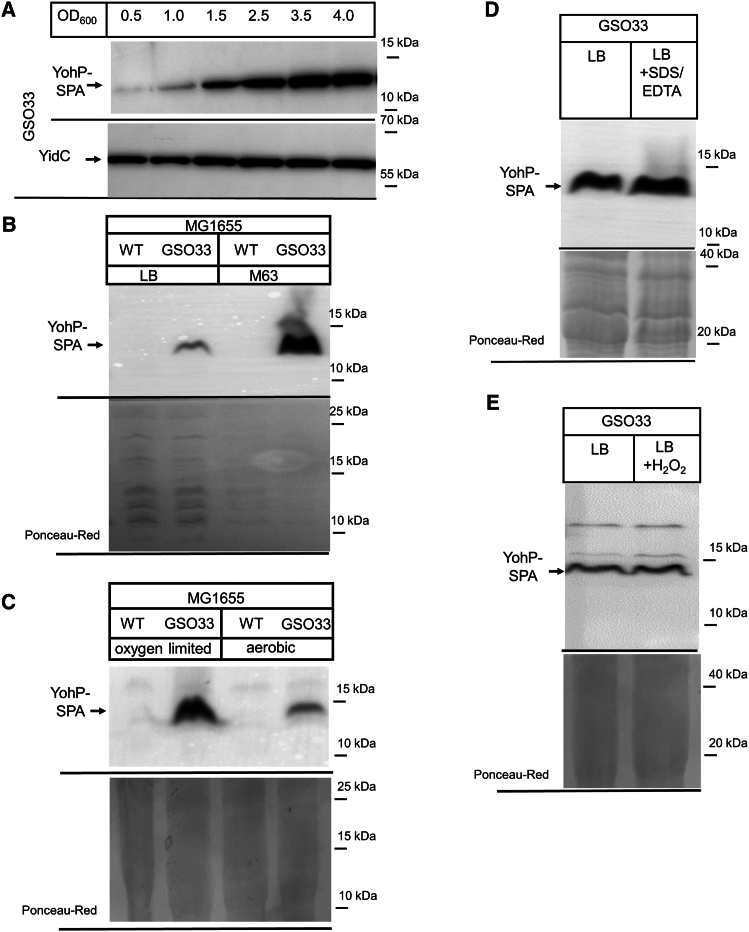
Native YohP levels increase when cells enter stationary phase or grow on minimal media (A) *E. coli* GSO33, an MG1655 variant containing a chromosomally SPA-tagged YohP under its native promoter (YohP-SPA), was grown on LB medium up to different ODs, and 2 × 10^8^ cells were precipitated with 5% trichloroacetic acid (TCA). After centrifugation, samples were denatured and separated on 16.5% Tris-Tricine SDS-PAGE followed by western transfer and immune detection with α-FLAG antibodies. The inner membrane protein YidC served as a loading control and was detected by polyclonal α-YidC antibodies. (B) MG1655 (WT) and its variant GSO33 were grown on LB or M63 minimal medium up to OD_600_ = 1.5, and cells were processed as above. Before blocking, the membrane was stained with Ponceau Red to control the amount of loaded protein. (C) MG1655 and GSO33 cells were grown on LB medium +0.2% glucose in either completely filled 2 mL Eppendorf tubes without shaking (oxygen-limited) or in 50 mL Falcon tubes with 5 mL LB + 0.2% glucose medium with shaking (aerobic). Cells were harvested at OD_600_ = 1.5 and processed as described above. (D) GSO033 cells were grown on LB medium, and at OD_600_ = 0.2 SDS (0.025%) and EDTA (1 mM) were added when indicated. After growth to OD_600_ = 1.5, cells were processed as above. (E) GSO33 cells were grown on LB medium and at OD_600_ = 0.2, 1 mM H_2_O_2_ was added. After growth to OD_600_ = 1.5 cells were processed as described above. Shown are representative blots of at least 3 independent biological replicates. See also Figure S2.

In screens that monitored the induction of multiple small proteins under different growth conditions, YohP was found to be up-regulated in stationary phase cells, in cells grown on minimal media, or upon treatment with SDS/EDTA or H_2_O_2_.^12^^,^^28^ This was validated in the current study by using the *E. coli* strain GSO33, which contained a C-terminally SPA-tagged *yohP* copy in the chromosome under the control of its native promoter.^12^ The SPA-tag consists of an 8 kDa calmodulin-binding peptide in addition to the triple-FLAG tag. Immune detection using α-FLAG antibodies confirmed that the YohP levels increased from early stationary phase (optical density [OD] 1.5) to late stationary phase (OD 4.0) (Figure 1A). Elevated YohP levels were also observed in cells grown on M63 minimal media (Figure 1B) or when cells were grown under oxygen-limited conditions (Figure 1C). Thus, the production of YohP appears to be primarily linked to nutrient limitation, which cells encounter when grown on minimal media, under oxygen limitation, or when they enter into stationary phase. However, we did not observe a significant change in the YohP levels when cells were treated with SDS/EDTA (Figure 1D), which induces cell envelope stress,^45^ or upon oxidative stress induced by H_2_O_2_ treatment (Figure 1E). The reason we were unable to reproduce the increased YohP production in SDS/EDTA- or H_2_O_2_-treated cells remains unclear. A possible explanation is the use of different antibodies and the inherently semi-quantitative nature of western blotting,^46^ particularly Dot-blots, which were used in the previous study.^28^

### YohP synthesis significantly alters the *E. coli* proteome

**Figure 2:**
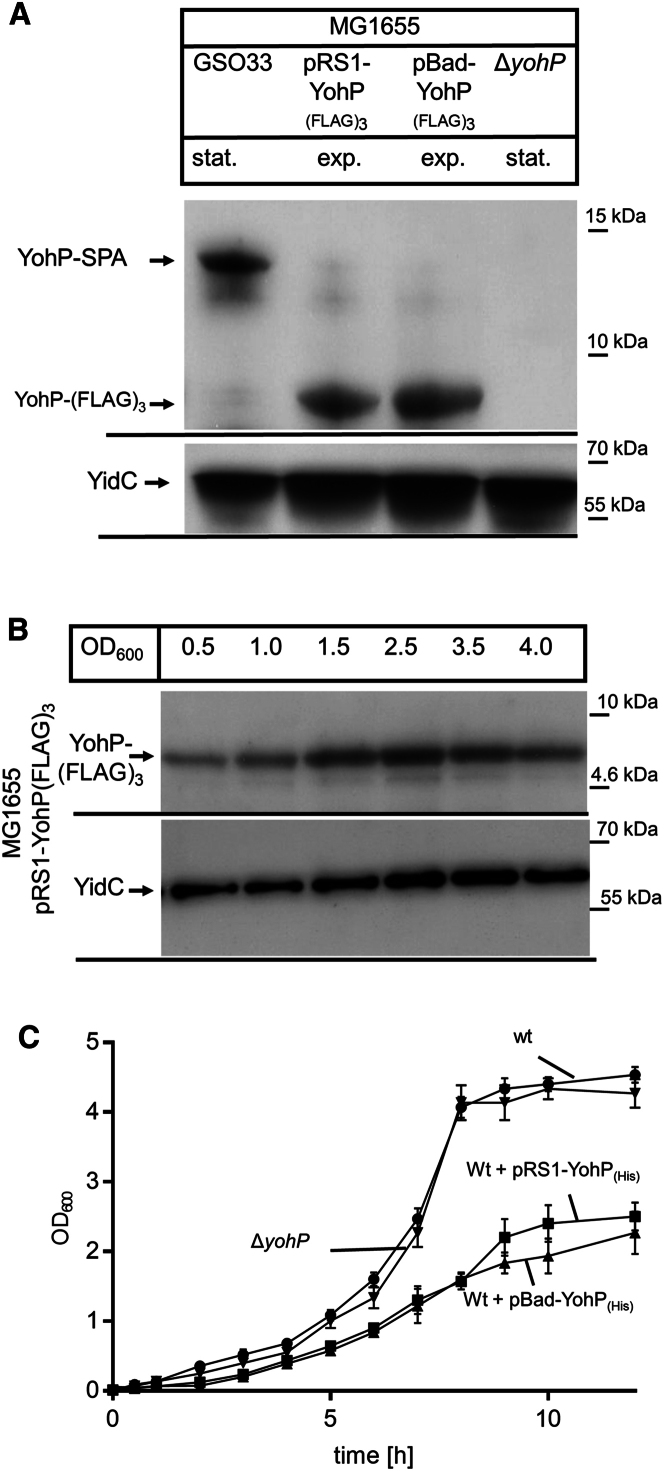
Premature production of YohP in exponential phase inhibits *E. coli* cell growth (A) *E. coli* MG1655 carrying the chromosomally tagged *yohP* copy (GSO33) was grown on LB medium up to OD_600_ = 2.5 (stationary phase, stat.). *E. coli* MG1655 cells containing the plasmid-encoded *yohP* variants in either vectors pRS1 or pBad24 were induced at OD 0.4 with either 1 mM IPTG or 0.2% arabinose for approximately 1–2 h (OD_600_ ∼1.0, exponential phase, exp.). The Δ*yohP* strain served as a control. 2 × 10^8^ cells were precipitated with 5% trichloroacetic acid (TCA). After centrifugation, samples were denatured and separated on 16.5% Tris-Tricine SDS-PAGE, followed by western transfer and immune detection with α-FLAG antibodies. The inner membrane protein YidC served as a loading control and was detected by polyclonal α-YidC antibodies. (B) MG1665 carrying pRS1-YohP(FLAG)_3_ was grown on LB medium up to the indicated OD_600_; YohP production was induced at OD_600_ = 0.2 with 1 mM IPTG. 2 × 10^8^ cells were precipitated with 5% trichloroacetic acid (TCA) and further processed as above. Representative blots of three independent experiments are shown. (C) The indicated strains were grown on LB medium, and YohP production from the plasmid was induced at OD_600_ 0.2 with either 1 mM IPTG or 0.002% arabinose. Cell growth was then monitored over time. Shown are the mean optical density readings of three independent biological experiments, and the error bars reflect the SD. See also Figure S2.

Since we were unable to completely reproduce the reported conditions for YohP production, we performed an unbiased proteome analysis for identifying proteins that were up- or down-regulated in response to the YohP levels. Nutrient limitation or the transition into stationary phase is associated with major proteomic rearrangements, which are largely coordinated by the σ-factor RpoS.^47^ RpoS regulates the transcription of approximately 1,000 genes in *E. coli*, representing around 25% of the entire genome.^48^^,^^49^ Thus, it would be difficult to determine the specific consequences of YohP production by simply monitoring proteomic changes in stationary *E. coli* cells. In addition to YohP, many other SMPs are induced in stationary phase,^12^^,^^28^ suggesting a significant degree of functional redundancy, which further complicates the characterization of YohP-specific effects in stationary-phase-grown cells. To enable more precise monitoring of the physiological consequences of YohP production, we used a plasmid-based approach that allowed induction of YohP expression already during the exponential growth phase. The validity of this approach depends on the plasmid-encoded YohP being expressed at levels comparable with those of the chromosomally encoded YohP during the stationary phase. Therefore, we compared two plasmid systems: the plasmid pRS1-YohP(FLAG)_3_ encodes *yohP* with a C-terminal triple-FLAG-tag under the control of the *T7*-promoter in the vector pRS1,^50^ and pBad24-YohP(FLAG)_3_, which contains the FLAG-tagged *yohP* under arabinose control in pBad24.^51^ The plasmid-encoded YohP levels during exponential growth phase were comparable with the chromosomally encoded YohP levels in mid-stationary phase (Figure 2A). The mass difference is explained by the presence of the 8 kDa calmodulin-binding peptide within the SPA-tag. Expression of the chromosomally encoded *yohP* copy is induced during stationary phase (Figure 1A), while the YohP levels of pRS1-YohP(FLAG)_3_-containing cells were largely constant (Figure 2B). The presence of the FLAG tag facilitated a direct comparison of the chromosomally and plasmid-encoded YohP levels. However, the triple FLAG tag almost doubles the mass of YohP and adds several charged amino acids, which potentially interferes with YohP function. In addition, the presence of either the FLAG tag or the SPA-tag prevented YohP dimerization (Figure S2A). Therefore, we also analyzed C-terminally His-tagged YohP variants, because previous data showed that the His-tag did not disrupt membrane insertion, dimerization, or membrane topology of YohP.^30^ The levels of YohP were also not influenced by the presence of the tag and were comparable with the YohP levels of GSO33 cells grown to stationary phase (Figure S2A).

Although YohP is mainly produced during stationary phase or on minimal media, the deletion of *yohP* via λ-red recombination^52^ did not result in significant growth defects in stationary phase (Figure 2C) or when cells were grown on M63 minimal media (Figures S2B and S2C). In contrast, producing YohP already during exponential phase reduced the growth rate, indicating that premature YohP production is toxic for *E. coli* cells (Figure 2C).

**Figure 3:**
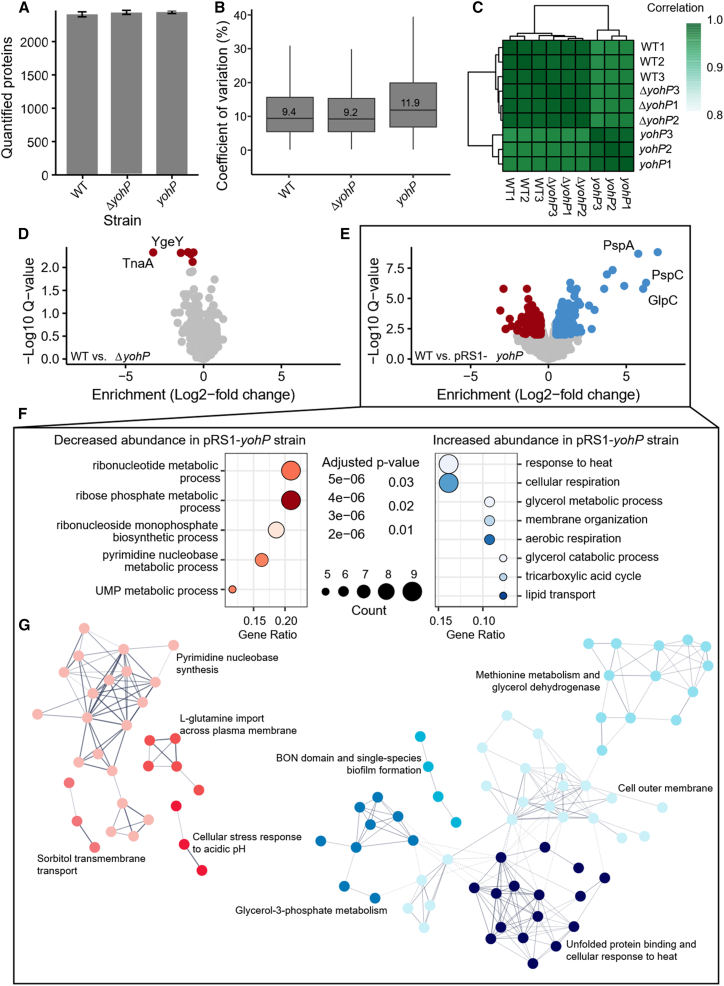
MS data provide insights into the role of YohP (A) Average number of quantified proteins in each measured sample group (*n* = 3); *yohP* refers to MG1655 carrying the plasmid pRS1-YohP_(His),_ and *E. coli* cells were grown as described in Figure 2. Error bars are represented in terms of SD. (B) Boxplot representation of the average coefficients of variation (%) for quantified proteins in each sample group (*n* = 3). (C) Correlation heatmap and clustering analysis of the measured samples. Correlation values in the legend are in terms of the coefficient of correlation (R). (D) Volcano plot for the comparison between wild-type *E. coli* and the *yohP*-deletion strain. Significantly differentially abundant proteins (q-value <0.05 and fold-change >2) are highlighted in red for decreased abundance in the mutant strain. (E) Volcano plot for the comparison between wild-type *E. coli* and the *yohP*-induction strain. Significantly differentially abundant proteins (q-value <0.05 and fold-change >2) are highlighted in red for decreased abundance in the mutant strain, and in blue for increased abundance. (F) Dot plots for the GO classification enrichment analysis for the comparison between wild-type *E. coli* and the *yohP*-induction strain. GO classifications for Biological Processes were selected. (G) String networks for the comparison between wild-type *E. coli* and the *yohP*-induction strain. Only differentially abundant proteins (q-value <0.05 and fold-change >2) were selected for the network analysis, and non-interacting nodes were removed. K-means clustering analysis of the network led to significant nodes (*p* < 0.05) grouped by different colors. See also Figures S3–S5).

To investigate the proteome remodeling deriving from *yohP* deletion or *yohP* induction (cells containing pRS1-YohP_(His)_), in comparison with wild-type *E. coli*, we conducted mass spectrometry (MS)-based proteomics in a data-independent acquisition mode (*n* = 3 per group). With our workflow, we quantified more than 2,500 proteins per sample (Figure 3A), achieving an average coefficient of variation of approximately 10% at the protein level (Figure 3B). This comprehensive dataset provided a solid foundation for a robust comparison of the *yohP* deletion and *yohP* induction strains against wild-type *E. coli*. Based on clustering analysis, we observed a high degree of similarity among all sample groups (R > 0.95) (Figure 3C), with the *yohP* induction strain showing a more prominent difference. We then carried out differential expression analysis using the DEqMS algorithm,^53^ implemented in the MS-DAP-R package,^54^ which accounts for the dependency of variance on the number of peptides used for quantification. This approach increases the number of quantifiable proteins without increasing false-positive rates, which is due to a more robust quantification of low-abundance proteins and proteins with a low number of peptide-spectrum matches. With the ability to quantify over 30,000 peptides per sample, our workflow enabled precise identification of significantly up-regulated and down-regulated proteins in the analyzed strains.

The comparison of the proteomes of the Δ*yohP* strain and the wild-type *E. coli* showed only a small number of proteins with decreased abundance in the absence of YohP and no protein with increased abundance (Figure 3D; Table S1). The strongest effect was observed for tryptophanase (TnaA), which is required for the production of the signaling molecule indole. The putative peptidase YgeY was also down-regulated. YgeY was identified in a screen for envelope biogenesis mutants,^55^ but so far, no clear functions have been assigned. Small effects were also observed for maltose transport proteins MalK and MalE, the ketol-acid reductoisomerase IlvC, and the L-asparaginase AnsB. A recent report demonstrates that MalK is down-regulated when the SOS DNA-damage response is induced.^56^ However, we did not see any growth impairment of the Δ*yohP* strain on maltose-McConkey-agar (Figure S2C), indicating that the Δ*yohP* strain can still utilize maltose as carbon source.

In contrast to the Δ*yohP* strain, the YohP-producing strain showed 80 proteins that were significantly up-regulated (Figure 3E; Table S2) and 52 down-regulated proteins (Figure 3E; Table S2). Based on gene ontology (GO) classification (Figure 3F), the most strongly up-regulated proteins corresponded to heat shock proteins/chaperones, proteins involved in cellular and aerobic respiration, in membrane organization and lipid transport, as well as glycerol-3-phosphate metabolism. The strongest up-regulations were observed for the phage-shock proteins PspA and PspC and for the anaerobic glycerol-phosphate dehydrogenase (GlpABCD). PspA, PspC, and GlpC have been shown to respond to membrane damage, induced e.g., by organic solvents or upon dissipation of the membrane potential.^57^^,^^58^ GO classification for cellular compartment revealed significant enrichments for proteins localized to the cell envelope and outer membrane (Figure S2).

Many proteins involved in nucleotide biosynthesis were down-regulated upon YohP production (Figure 3F). This included carbamoylphosphate synthase (CarB), which initiates pyrimidine biosynthesis, aspartate transcarbamylase (PyrB), which catalyzes the second step of pyrimidine biosynthesis and many more proteins involved in pyrimidine synthesis (PyrD, PyrC, Upp). In addition, the uracil transporter UraA, the cytosine transporter CodB, and the glutamine transporter GlnHPQ were down-regulated. GlnHPQ constitutes an ABC transporter required for high-affinity uptake of glutamine,^59^ which serves as substrate for the CarB reaction. Some proteins involved in purine metabolism, such as GMP-reductase GuaC, or xanthine-guanine phosphoribosyltransferase Gpt, were also down-regulated. In summary, these down-regulations most likely reduce the *de novo* synthesis and salvage pathways of pyrimidine nucleotides, and, to a lesser extent, purine nucleotides.

The production of YohP also reduced the levels of some regulatory proteins, such as AppY, GadE, YfeC, or GlnG (NtrC). AppY regulates genes of the anaerobic energy metabolism, such as the hydrogenase-I (*hyaABCDEF*), which we also found to be down-regulated. Furthermore, both AppY and GadE control genes involved in acid resistance^60^^,^^61^ and both have been linked to the SOS DNA damage control.^56^ YfeC acts as a dual regulator that is involved in regulating DNA replication, translation, and cell envelope biogenesis.^62^ Finally, NtrBC constitutes a two-component system that controls the transcriptional response to nitrogen starvation and regulates amino acid metabolism.^63^

We have also observed a high degree of interaction between the differentially expressed proteins. Network analysis using STRING^64^ revealed that the protein-protein interaction (PPI) enrichment *p*-value for the down-regulated proteins in the YohP-overexpressing strain was smaller than 1e^−16^. The same degree of PPI enrichment was observed for the up-regulated proteins. This means that the degree of interaction of the up- and down-regulated proteins is significantly higher than what would be expected for a random set of proteins, indicating that they are biologically connected (Figure 3G). We have also verified by k-means cluster analysis similar biological function enrichments to what we have observed in GO-terms analysis (Figures 3G; S3, S4, and S5). Furthermore, we investigated the interactions between up- and down-regulated proteins and observed similar PPI enrichment *p*-values (Figure S4). These data show that there are interactions between proteins differentially regulated in the YohP-producing strain. Examples include GadE (down-regulated), YdeP and AdiY (up-regulated), involved in acid resistance; GalE (down-regulated) and CpsG (up-regulated), involved in galactose metabolism; and GltS (up-regulated) and GlnQ (down-regulated), involved in glutamine/glutamate transport.

In summary, the deletion of *yohP* had only a minor effect on the *E. coli* proteome under the conditions tested and mainly reduced the amount of tryptophanase. The small changes in the proteome potentially also explain why no detectable phenotypes have been observed so far for the Δ*yohP* strain. In particular, there was no growth phenotype during transition into stationary phase (Figure 2C) or when cells were grown on M63 minimal media (Figures S2B and S2C), conditions that both induced *yohP* expression (Figure 1).

In contrast, the premature expression of YohP during exponential phase caused a multi-layered change in the protein composition, with the most striking effects on proteins involved in acid response, membrane damage, and nucleotide biosynthesis. This is also reflected by the reduced growth rate of the *yohP*-producing strain (Figure 2C).

### The absence of YohP reduces the survival rate of *E. coli* under stress conditions

**Figure 4:**
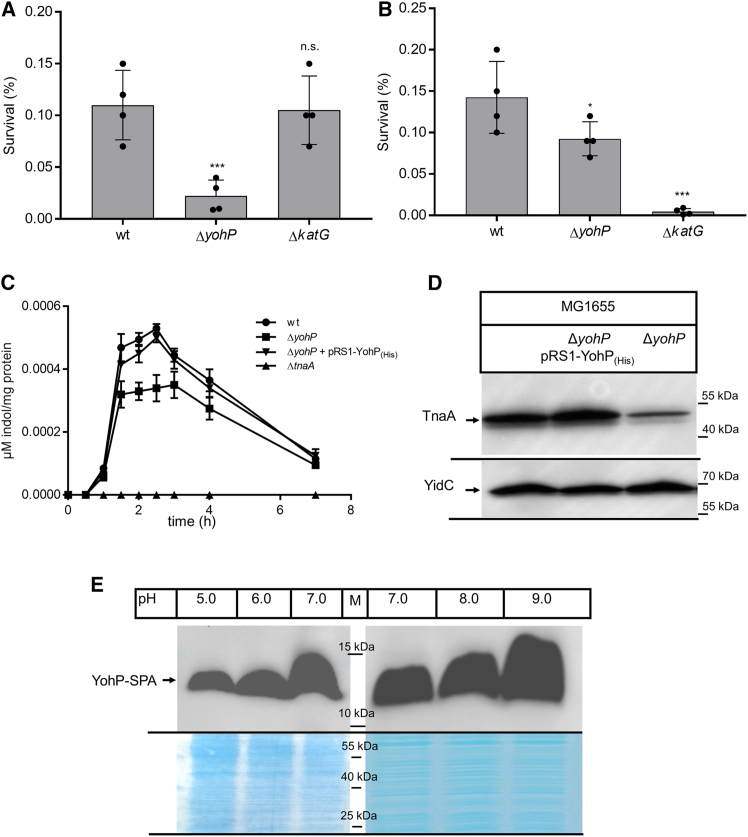
*E. coli* cells lacking YohP show increased sensitivity toward ciprofloxacin and H_2_O_2_, but not to acid stress (A) The survival rate of the indicated *E. coli* cells in the presence of ciprofloxacin was determined by treating cells during exponential phase for 6 h at 37 °C with 5 μg/ml ciprofloxacin. The *E. coli* strain Δ*katG* lacks catalase G and was used as a control.^68^^,^^69^ The number of viable cells before and after treatment was determined using the QUANTOM TxTM Microbial Cell Counter and the QUANTOM Viable Cell Staining Kit and the relative survival rate was calculated. The bars represent the mean of four biological replicates, and the SD is indicated by error bars. Dots show the individual data points. (B) As in A, but cells were treated for 30 min at 37 °C with 2 mM H_2_O_2_. To determine the significance of the results, the *p* value was calculated using the “unpaired, two-tailed t test” of the program Graph Pad PRISM 6 or the one-way ANOVA analyses and Turkey honest significance test using the values in the wild-type strain as reference. The P-values are depicted as asterisks (∗) above the graphs as follows: n.s. = *p* > 0.05; ∗ = *p* ≤ 0.05; ∗∗∗ = *p* ≤ 0.005. (C) *E. coli* cells were grown on tryptone broth, and the secreted indole in the supernatants was quantified after different times of incubation using the Kovacs reagent. Shown are the mean values (*n* ≥ 3), and the error bars represent the SD. YohP production was induced at OD_600_ = 0.2 with 1 mM IPTG. (D) *E. coli* cells were grown as in C up to OD 0.8–1.0, and 2 × 10^8^ cells were processed as in Figure 1 and further analyzed with α-TnaA antibodies. Antibodies against YidC served as a loading control. (E) *E. coli* GSO33 cells were grown on LB medium adjusted to different pH values up to OD_600_ ∼1.5, and 2 × 10^8^ cells were processed as in Figure 1. The lower panel corresponds to a Coomassie blue-stained part of the gel as a loading control. Shown are representative blots of three independent experiments. See also Figure S6.

It was recently shown that some SMPs influence the bacterial stress resistance by regulating the activity of membrane transporters.^16^^,^^65^ We therefore analyzed whether the Δ*yohP* strain showed increased sensitivity to antibiotics, such as ciprofloxacin, or to oxidative stress. This was indeed the case, and the Δ*yohP* strain showed a reduced survival rate when treated with ciprofloxacin (Figure 4A). As a control, we also tested the survival rate of a strain lacking the catalase KatG, because some antibiotics were suggested to induce oxidative stress.^66^^,^^67^ However, the survival rate of the Δ*katG* was comparable with the survival rate of the wild-type strain (Figure 4A). The Δ*yohP* strain also showed a reduced survival rate when treated with H_2_O_2_ to induce oxidative stress, but this was less pronounced than in the Δ*katG* strain (Figure 4B). Thus, the absence of YohP is associated with increased ciprofloxacin and H_2_O_2_ sensitivity.

Bacterial antibiotic resistance has been linked to indole signaling, which is suggested to influence antibiotic uptake,^70^ but there are conflicting results as to whether indole increases or reduces antibiotic resistance.^71^^,^^72^ Still, indole signaling is likely impaired in the Δ*yohP* strain due to the reduced TnaA levels (Figure 3D). We therefore determined the indole levels in the Δ*yohP* strain. The indole levels were initially monitored in cells grown on phosphate-rich medium (INV medium), which was also used for the proteomics approach. However, under those conditions, indole was not detectable. We therefore switched to tryptone broth, which has previously been used for monitoring indole levels. Indole was detectable in wild-type cells but not in the Δ*tnaA* strain (Figure 4C). Indole formation in wild-type cells reached a maximum at late exponential phase and then declined. In the absence of YohP, the indole production was reduced but not completely diminished (Figure 4C), which is in line with the reduced TnaA levels shown in the proteome analysis of the Δ*yohP* strain. In Δ*yohP* strains carrying the plasmid-borne *yohP* copy, the indole levels were comparable with those in the wild type. We also monitored the TnaA levels by immune detection and observed reduced but not completely diminished TnaA levels in the Δ*yohP* strain (Figure 4D), supporting the decreased but still detectable indole production in this strain.

Whether the small reduction of the indole levels in the absence of YohP was responsible for increased stress sensitivity was further analyzed by monitoring the acid resistance of *E. coli*. Indole production has been linked to bacterial acid resistance, but as for the antibiotic resistance there are conflicting results as to whether it inhibits^73^ or stimulates^74^ the acid resistance system. A possible link between the YohP levels and acid resistance is supported by the down-regulation of the transcriptional regulators AppY and GadE, which are involved in acid resistance,^60^^,^^61^ and of glutamine ABC transporter GlnHPQ, which also influences acid resistance.^75^ For analyzing a potential link between YohP expression and acid resistance, the GSO33 strain, containing the chromosomally tagged *yohP* copy, was grown in M63 medium at different pH values and the YohP levels were determined by western blotting using α-FLAG antibodies. This revealed that increasing the pH from 5.0 to 9.0 caused an increase in the YohP levels (Figure 4E), demonstrating that YohP expression is pH regulated. However, we did not observe any specific growth defect of the Δ*yohP* or *yohP*-overproducing strains at different pH values (Figure S6), suggesting that YohP is not a major factor for controlling *E. coli* growth at different pH values.

In summary, Δ*yohP* cells show increased sensitivity to ciprofloxacin and H_2_O_2_ but not to acid stress. However, it is currently unknown whether this is related to changes in indole signaling, because the reduction of the intracellular indole level is rather small and reduced indole concentrations have been mainly associated with increased not reduced resistance toward antibiotics.^70^^,^^71^^,^^72^^,^^76^

### YohP influences membrane composition and fluidity

**Figure 5:**
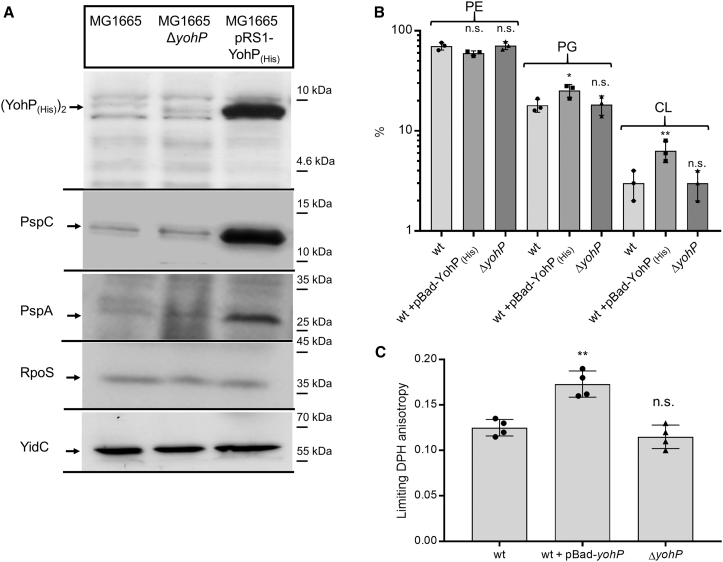
YohP causes membrane damage (A) *E. coli* cells were grown on LB medium up to an OD of 1.3, and 2 × 10^8^ cells were processed as in Figure 1 and the membrane was decorated with the indicated polyclonal antibodies, or, in the case of YohP, with monoclonal α-His antibodies. YohP production was induced with 1 mM IPTG at OD_600_ = 0.4. Representative blots of three independent experiments are shown. (B) Lipidomic analyses of isolated inner membrane vesicles (INVs) derived from the indicated strains. The extraction, analysis, and quantification were performed by Lipotype GmbH, Dresden, Germany, as described in STAR Methods. Shown are the percentage of the main *E. coli* lipids (PE, phosphatidylethanolamine; PG, phosphatidylglycerol; and CL, cardiolipin). The individual values are indicated by dots, the mean values by the columns, and the SD by the error bars (*n* = 3). (C) Membrane order in INVs of the indicated strains (1 mg protein/ml) was monitored in terms of the limiting fluorescence anisotropy of the probe 1.6-Diphenyl-1,3,5-hexatriene (DPH), i.e., the value approached by the anisotropy decay for infinite time after excitation. Shown are scatterplots of four independent experiments, with the mean value indicated by the column and the SD by the error bar. To determine the significance of the results, the P-value was calculated using the “unpaired, two-tailed t test” of the program Graph Pad PRISM 6 or the one-way ANOVA analyses and Turkey honest significance test using the values in the wild-type strain as reference. The P-values are depicted as asterisks (∗) above the graphs as follows: n.s. = *p* > 0.05; ∗ = *p* ≤ 0.05; ∗∗ = *p* ≤ 0.01. See also Figure S7.

The proteomic data showed the up-regulation of PspC, PspA, and GlpC in the MG1655 pRS1-YohP_(His)_ strain, which is indicative of membrane damage. This was further confirmed by immune detection, which demonstrated the up-regulation of PspC and PspA in the MG1655 pRS1-YohP_(His)_ strain (Figure 5A). In agreement with previous data,^30^^,^^32^ the presence of the His tag does not prevent YohP dimerization and, therefore, YohP is detected at approximately 9 kDa (Figure 5A). The levels of the major stress-responsive σ-factor RpoS were also analyzed for excluding secondary effects, but there were no significant differences between the strains.

The structure and stability of the bacterial membrane are influenced by its lipid composition.^77^ To investigate whether YohP production affects this composition, we employed a lipidomics approach using sucrose-gradient-purified inner membrane vesicles (INVs). The most striking difference was a more than 2-fold increase in the cardiolipin content in the pRS1-YohP_(His)_-containing strain (Figure 5B). The diacylglycerol (DAG) and phosphatidic acid contents also increased in this strain (Figure S5), but these lipids generally accounted for less than 2% of the total lipid content.^78^ In the Δ*yohP* strain, the lipid content was comparable with the wild type (Figure 5B).

Both DAG and cardiolipin influence membrane stability.^77^ DAG has been suggested to seal membranes,^79^ while an increase in cardiolipin is observed when the ΔpH is reduced due to uncouplers.^80^ For monitoring the effect of YohP on the physicochemical properties of membranes, fluorescence anisotropy using the fluorescent probe diphenylhexatriene (DPH)^81^ was employed. In comparison with wild-type INVs, INVs from the YohP-producing strain showed increased limiting anisotropy, indicative of increased lipid order and reduced membrane fluidity (Figure 5C). The rotational correlation times of DPH did not differ significantly (not shown). In contrast, INVs of the Δ*yohP* strain showed values comparable with wild-type INV. Considering that cardiolipin is expected to increase membrane fluidity,^77^ the increased rigidity of the membrane is likely a direct effect of the presence of YohP in the membrane rather than an indirect effect via changes in the lipid composition.

### The proton motive force is reduced in the presence of YohP

**Figure 6:**
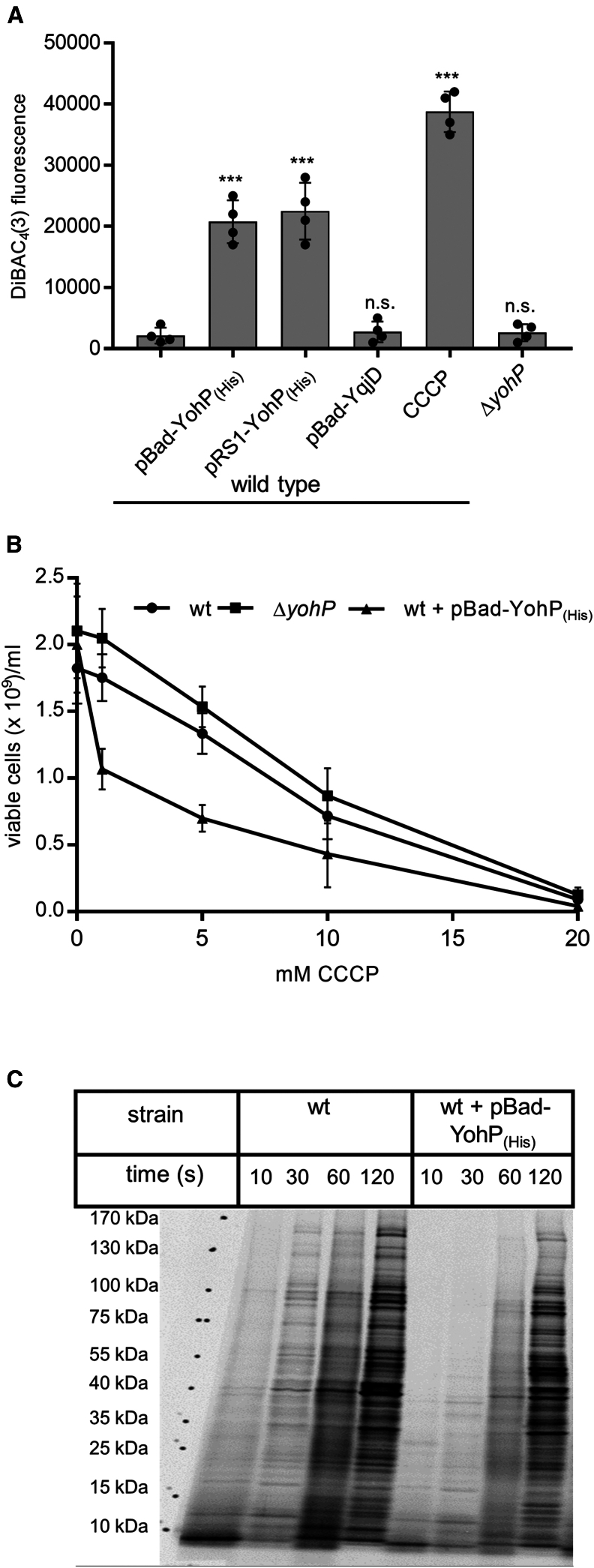
YohP dissipates the membrane potential (A) The indicated strains were grown on LB medium, and YohP production from the plasmid was induced at OD 0.4 with 0.2% arabinose or 1 mM IPTG, followed by further growth at 37°C for 2 h. Aliquots of 8 × 10^8^ cells were collected from the samples, washed once with sterile PBS, and further incubated for 30 min at 37°C with either PBS or 5 mM CCCP (Carbonyl-cyanide-*m*-chlorophenyl hydrazine, dissolved in DMSO). Samples were further processed as described in STAR Methods. DiBAC4(3) fluorescence emission at 530 nm was measured in 96-well plates after excitation at 485 nm. The individual values are shown by dots, and the columns indicate the mean values. SD is indicated by the error bars. Statistical analyses were performed on four independent biological replicates as described in Figure 5. n.s. = p > 0.05; ∗∗∗ = p < 0.005. (B) Cells were grown as in A and treated with different CCCP concentrations or with DMSO as a control for 1 h at 37°C. The number of viable cells was then counted using the QUANTOM TxTM Microbial Cell Counter and the QUANTOM Viable Cell Staining Kit. Shown are the mean values of three independent biological replicates with two technical replicates each (*n* = 6). The error bars indicate the SD. (C) Global protein synthesis was monitored in cells growing on M63 minimal medium supplemented with ^35^S-labeled methionine and cysteine. YohP production from the plasmid was induced at the start of the experiment by adding 0.2% arabinose. At the indicated time points, 100 μL culture was directly precipitated with TCA and separated by SDS-PAGE. Radioactively labeled proteins were visualized by autoradiography. Shown is a representative image of three independent experiments. See also Figure S7.

The increase of PspA and PspC is frequently linked to the dissipation of the membrane potential.^82^^,^^83^ For monitoring the effect of YohP on the membrane potential, cells were stained with the slow-responsive, potential-sensitive dye DiBAC_4_(3) (bis-(1,3-dibutylbarbituric acid) trimethine oxonol). DiBAC_4_(3) enters depolarized cells and binds to intracellular proteins and to membranes. Increased depolarization results in increased influx of the anionic dye and increases fluorescence. Thus, DiBAC_4_(3) fluorescence at 530 nm after excitation at 485 nm is proportional to the membrane depolarization. This was confirmed by treating *E. coli* wild-type cells with 5 mM of the uncoupler CCCP (cyanide-*m*-chlorophenyl hydrazine). In CCCP-treated cells, a strong increase in DiBAC_4_(3) fluorescence was observed (Figure 6A). Increased fluorescence was also observed in *yohP*-expressing cells, independently of whether YohP was expressed from pRS1 or pBad24, while the Δ*yohP* strain showed a fluorescence comparable with the wild type. As an additional control, we also analyzed YqjD-producing cells. YqjD is a small C-tail anchored membrane protein that, like YohP, contains a single transmembrane domain and also a high propensity for dimer formation.^84^ However, YqjD-producing cells did not show increased fluorescence, demonstrating that YohP insertion into the *E. coli* membrane specifically impairs the proton motive force (pmf). The YohP-induced dissipation of the membrane potential is expected to increase the cellular sensitivity to protonophores such as CCCP. Consistent with this hypothesis, YohP-expressing cells demonstrated decreased viability upon CCCP treatment relative to wild-type and Δ*yohP* strains (Figure 6B).

The partial dissipation of the pmf should reduce the metabolic activity of the YohP-producing cells, and this was monitored in a metabolic labeling experiment. Protein synthesis is one of the most energy-consuming processes in the bacterial cell^85^ and provides a reliable readout for metabolic activity. Wild-type cells and cells producing YohP from the plasmid pBad-YohP_(His)_ were grown on minimal media in the presence of ^35^S-labeled methionine and cysteine to monitor the global protein synthesis rate over time. This demonstrated that the protein synthesis rate of *yohP*-expressing cells was significantly reduced in comparison with wild-type cells (Figure 6C). This validates that the production of YohP dissipates the membrane potential, which in turn reduces the efficiency of energy-consuming processes.

### YohP production is linked to the stringent response

**Figure 7:**
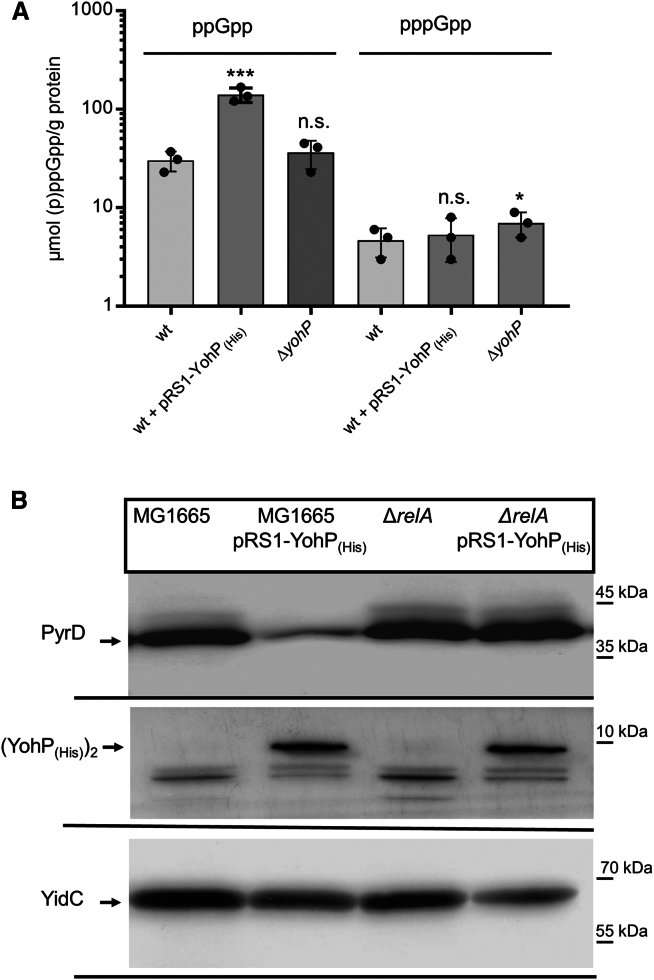
YohP production induces the stringent response (A) *E. coli* cells were grown on LB medium up to OD_600_ = 0.8, and *yohP* expression was induced at OD_600_ = 0.4 with 1 mM IPTG. Subsequently, 20 × 10^8^ cells were lysed and extracted as described in STAR Methods. Samples were spiked with heavy [^15^N] (p)ppGpp standards before extraction. (p)ppGpp levels in the extracted samples were determined by MS-coupled capillary electrophoresis. The assay was performed with three biological replicates and three technical replicates each (*n* = 9). The mean values of the three technical replicates are shown by dots, and the mean values of the three biological replicates is indicated on the columns. The error bars reflect the SD. (B) The *E. coli* strains were grown as in A, and 2 × 10^8^ cells were TCA precipitated and separated by SDS-PAGE, followed by immune detection using α-PyrD, α-FLAG, and α-YidC antibodies. Shown are representative blots of three independent experiments. To determine the significance of the results, the P-value was calculated using the “unpaired, two-tailed t test” of the program Graph Pad PRISM 6 or the one-way ANOVA analyses and Turkey honest significance test using the values in the wild-type strain as reference. The P-values are depicted as asterisks (∗) above the graphs as follows: n.s. = *p* > 0,05; ∗ = *p* ≤ 0,05; ∗∗∗ = *p* ≤ 0,005. See also Figure S7.

One striking effect of YohP production is the down-regulation of several enzymes involved in pyrimidine biosynthesis. Due to the high energy demand of their *de novo* synthesis, purine and pyrimidine biosynthesis in *E. coli* is strictly regulated.^86^^,^^87^ One mechanism that coordinates nucleotide biosynthesis with nutrient availability in bacteria is the stringent response.^8^^,^^88^^,^^89^^,^^90^ The stringent response describes the accumulation of the stress-signaling molecules pppGpp and ppGpp, which induce multi-layered changes in cellular metabolism, including changes in transcription, translation, and protein targeting.^31^^,^^32^^,^^91^^,^^92^ For analyzing whether YohP production influences the stringent response, the (p)ppGpp levels in cell extracts were determined by capillary electrophoresis combined with MS^93^^,^^94^ (Figure 7A). This approach revealed 4-fold increased ppGpp levels in the YohP-producing strain compared with the wild-type strain. The pppGpp levels in all three strains were significantly lower than the ppGpp levels, which is in line with published data^95^ and mainly caused by GppA, which converts pppGpp into ppGpp.^96^ The Δ*yohP* strain showed a small increase in the pppGpp levels, but whether this is the result of the reduced TnaA and/or indole levels needs to be further analyzed. Thus, the induction of *yohP* causes an up-regulation of ppGpp, which in turn could repress pyrimidine biosynthesis. This was further validated by expressing pRS1-YohP_(His)_ in a strain that lacks RelA, the key enzyme for (p)ppGpp production in *E. coli*.^8^ As a readout for enzymes of the pyrimidine biosynthesis, we monitored the levels of the dihydroorotate dehydrogenase PyrD. The PyrD levels in strain MG1665 containing pRS1-YohP_(His)_ were reduced in comparison with the MG1665 wild type, confirming the results from the MS. Importantly, there was no significant difference in the PyrD levels between the Δ*relA* strain and the Δ*relA* strain containing pRS1-*yohP* (Figure 7B), which is explained by the reduced amounts of (p)ppGpp that are synthesized when RelA is missing.^97^ The inner membrane protein YidC served as a loading control in these experiments. In conclusion, the down-regulation of enzymes involved in pyrimidine biosynthesis is likely the consequence of the YohP-induced stringent response.

## Discussion

Although technical improvements have significantly advanced the identification of SMPs in bacterial proteomes,^98^ their functional characterization remains a bottleneck for understanding their role in bacterial physiology.^25^^,^^99^^,^^100^^,^^101^^,^^102^^,^^103^ In the current study we employed a multidisciplinary approach to investigate and gain insights into the function of YohP in *E. coli.* Our data demonstrate that YohP insertion into the *E. coli* membrane causes membrane damage that leads to the dissipation of the proton motive force. This, in turn, induces significant changes in the proteome, stimulates the stringent response, and converts cells into a state of metabolic silence.

One important observation of our study is that the deletion of *yohP* in *E. coli* increases the ciprofloxacin sensitivity and the sensitivity toward oxidative stress. However, under conditions that induce YohP expression in wild-type cells—such as stationary phase or growth in minimal media—the Δ*yohP* strain showed no detectable growth phenotype. This is likely related to a possible functional redundancy among different SMPs,^12^^,^^14^^,^^27^^,^^98^ although we did not observe a significant up-regulation of other SMPs upon deletion of *yohP* in our proteomic analyses. In general, the deletion of *yohP* had only a minor effect on the *E. coli* proteome. Only TnaA showed a significant down-regulation in the absence of YohP, and although indole, the product of the TnaA reaction, has been linked to stress signaling and resistance in bacteria,^72^^,^^73^^,^^74^ indole production was only slightly reduced in the *E. coli* Δ*yohP* strain. This likely reflects the fact that TnaA was still detectable by immune detection in the Δ*yohP* strain. Besides TnaA, only YgeY showed an obvious down-regulation upon *yohP* deletion. YgeY is a putative peptidase that was identified in a genetic screen for mutants defective in cell envelope biogenesis.^55^ STRING analyses^64^ also link it to the salvage pathway of purines, but its exact function is still unknown.

Contrary to the minor proteomic effects of the Δ*yohP* strain, the premature YohP production during exponential phase results in major proteomic rearrangements. The highest up-regulation was observed for proteins that sense membrane damage, such as PspA, PspC, and GlpC.^57^^,^^104^ PspC, together with the transcriptional activator PspF, activates the phage-shock response, which stabilizes the bacterial membrane and protects it against envelope stress.^82^^,^^83^^,^^104^ PspA is suggested to sense proton leakage through damaged membranes and to reduce further leakage by forming a carpet-like seal below the membrane.^83^^,^^105^ The up-regulation of the phage-shock response is in line with the impaired membrane potential and the observed changes in membrane composition and fluidity in response to YohP insertion. PspF also activates the alternative sigma factor σ^54^ (RpoN),^106^ which responds to nitrogen starvation and regulates more than 130 genes involved in nitrogen metabolism, the stringent response, and stress signaling.^107^^,^^108^ This potentially explains the increased production of the alarmones (p)ppGpp upon YohP insertion. RelA-dependent (p)ppGpp production is typically induced by uncharged tRNAs as a consequence of amino acid starvation.^109^^,^^110^ Amino acid uptake in *E. coli* involves secondary transporters that use the electrochemical gradient as an energy source, but their activity is compromised when the membrane potential is dissipated.^111^ Amino acid starvation upon YohP-induced membrane damage will further stimulate the stringent response and potentially explains the differential expression of many enzymes involved in amino acid metabolism that we observe in our study. A major target of (p)ppGpp is the machinery required for ribosome biogenesis and translation,^8^^,^^85^ which rationalizes the reduced global protein synthesis when YohP is expressed. However, our proteome analysis did not show any indication for reduced ribosomal protein levels, except for RpsU (bS21), which is required for translation initiation and transcriptionally inhibited by ppGpp.^112^ But in general, the (p)ppGpp levels upon YohP production apparently do not reach levels that are sufficient to completely inhibit ribosome biogenesis.^8^^,^^85^^,^^113^

Several enzymes involved in the assembly of the outer membrane and the peptidoglycan biosynthesis are also up-regulated upon YohP production, and the up-regulation of some of these proteins (e.g., MlaB, LptE, MepM) has been linked to increased resistance phenotypes.^114^^,^^115^^,^^116^^,^^117^ Thus, it is tempting to speculate that the YohP-linked changes in the cell envelope promote the overall survival under stress conditions. However, in contrast to published reports,^28^ we did not observe an up-regulation of YohP upon SDS/EDTA-induced cell envelope stress.

Many of the down-regulated proteins are linked to amino acid, ribose phosphate, and nucleotide metabolism, which are all coordinated by the stringent response.^86^^,^^118^ The inhibition of purine nucleotide biosynthesis by (p)ppGpp has been reported before^89^^,^^119^ and is also visible in our study. In contrast, less is known about a possible impact of (p)ppGpp on pyrimidine metabolism. However, enzymes involved in pyrimidine biosynthesis were identified as potential (p)ppGpp targets in ppGpp-capture studies.^92^^,^^119^ Furthermore, transcription of *pyrD* was shown to be strongly inhibited in the presence of ppGpp,^120^ which explains why the PyrD levels are reduced upon YohP-induced ppGpp accumulation. Thus, the induction of the stringent response as a consequence of YohP-induced membrane damage leads to an overall reduction of the nucleotide pool.

Many of the effects seen upon YohP insertion into the membrane are detrimental to the cell, which explains the reduced growth rate. It is important to emphasize that endogenous YohP levels are quite high during stationary phase and match those of the plasmid-encoded YohP (*c.f.* Figure 2). The major difference between the endogenous YohP production and the experimental system used here is that our system monitored the effect of premature YohP production in exponentially growing cells. This still raises the question about the advantage of producing an apparently inhibitory protein when *E. coli* enters stationary phase. It could be related to the increased stress resistance of metabolically silent cells, which cumulates in so-called persister cells, which can escape the effects of many antibiotics by entering a state of metabolic dormancy.^40^^,^^121^^,^^122^ Nonheritable antibiotic resistance is also facilitated by a reduced pmf,^123^ and the purpose of producing YohP during stationary phase could be to reduce metabolic activity as a trade-off for increased resistance against non-favorable conditions. Reducing metabolic activity is a common strategy of bacteria during stationary phase and is well exemplified by ribosome hibernation factors, which transiently inactivate ribosomes and reduce global protein synthesis.^84^^,^^113^^,^^124^

In conclusion, our data indicate that the primary effect of YohP production in *E. coli* is to induce a non-lethal membrane damage, which in turn leads to a partial metabolic silencing that ultimately protects cells against further damage and helps them to save cellular resources when nutrients are scarce.

### Limitations of this study

Our multidisciplinary study analyzed the consequences of a premature YohP production already during exponential phase. However, we can currently not exclude that the consequences are different under native *yohP* expression conditions, e.g., when cells enter stationary phase or grow at elevated pH values. Thus, in a next step, it would be important to perform the proteomic analyses under the native conditions. This will potentially identify additional proteins that are differentially expressed when *yohP* is deleted. Our study revealed a down-regulation of TnaA, but we observed only a small reduction in the indole levels. This needs to be further explored by more sensitive detection methods, which could also include metabolic flux experiments to obtain a more global view on the metabolic silencing. Finally, considering that *E. coli* contains several YohP-like SMPs, it remains to be seen whether they induce similar effects.

## Resource availability

### Lead contact

Further information and requests for resources and reagents should be directed to and will be fulfilled by the lead contact, Hans-Georg Koch (hans-georg.koch@biochemie.uni-freiburg.de).

### Materials availability

All materials generated in this study will be available upon request.

### Data and code availability

• The proteomics data have been deposited at the ProteomeXchange Consortium via the PRIDE 137 partner repository with the dataset identifier PXD063229.
• The paper does not report original code.
• Original western blot images will be shared by the lead author upon request.
• Any additional information required to reanalyze the data reported in this paper is available from the lead author upon request.

## Acknowledgments

We are grateful to Dr. Gisela Storz, NIH Bethesda, USA, for providing the *E. coli* GSO33 strain and to Dr. Dirk Schneider, Johannes Gutenberg University Mainz for providing the PspA antibody. This work was supported by grants from the German Science Foundation (DFG) via the Priority Program SPP 2002 to J.D.L. and P.H.C.F. (JL3542/1) and H.-G.K. (KO2184/9), the RTG 2202 to H.H. and H.-G.K. (project ID 278002225), and the SFB1381 to H.-G.K. (Project-ID 403222702). In addition, support by the 10.13039/501100001659DFG via project IDs 450216812 and 409673687 is greatly acknowledged. P.H.C.F. and J.D.L. acknowledge support from the 10.13039/501100004189Max Planck Society. This project has received funding from the 10.13039/501100000781European Research Council under the European Union’s Horizon 2020 research and innovation program (grant agreement no. 864246, to H.J.J.).

## Author contributions

Conceptualization, H.-G.K., J.D.L.; investigation, A.N., N.M., M.M., E.S., I.P., I.C., P.H.C.F.; visualization, A.N., N.M., I.P., I.C., H.-G.K.; funding acquisition, H.-G.K., P.H.C.F., J.D.L., H.H.; supervision, H.-G.K., J.D.L., H.J.J., H.H.; writing, A.N., N.M., M.M., H.J.J., H.H., P.H.C.F., J.D.L., H.-G.K. All authors have read and commented on the manuscript.

## Declaration of interests

The authors declare that they have no conflicts of interest with the content of this article.

## Declaration of generative AI and AI-assisted technologies in the writing process

During the preparation of the manuscript, the authors used ChatGPT-4o for language editing purposes only. After using this tool, the authors reviewed and edited the content as needed and take full responsibility for the content of the publication.

## STAR★Methods

### Key resources table

**Table:** 

REAGENT or RESOURCE	SOURCE	IDENTIFIER
**Antibodies**		

mouse α-Flag M2	Merck, Darmstadt, Germany	Cat#F3165
rabbit α-PyrD	Cusabio via Biozol, Hamburg, Germany	Cat#CSB-PA812570XA01FQR
rabbit α-PspC	Cusabio via Biozol, Hamburg, Germany	Cat#CSB-PA365328XA01SZB
rabbit α-PspA	Dirk Schneider, Univ. Mainz	N.A.
mouse α RpoS	Biolegend, San Diego, USA	Cat#663703
rabbit α-YidC	Koch et al.^125^ Koch et al.^126^	N.A.
rabbit α-TnaA (polyclonal)	AssayPro, St.Charles, USA	Cat#33517-05111
mouse α-His	Invitrogen	Cat#MA1-21315
goat α rabbit IgG (KPL)	SeraCare, Milford, USA	Cat#5450-0010
goat α mouse IgG (KPL)	SeraCare, Milford, USA	Cat#5450-0011

**Bacterial and virus strains**		

*E. coli* MG1655	Blattner et al., 1997	N.A.
*E. coli* BW25113	Datsenko & Wanner,^52^	N.A.
*E. coli* MG1655 (with chromosomally tagged *yohP*;GSO333)	Dr. Gisela Storz (NIH, USA)	N.A.
*E. coli* (Δ*relA*)	KEIO Collection	JW2755 Cat#OEC4987-213606232
*E. coli* (Δ*tnaA*)	KEIO Collection	JW3686 Cat#OEC4987-200828669
*E. coli* (Δ*katG*)	KEIO Collection	JW3914 Cat#OEC4987-213605122

**Chemicals, peptides, and recombinant proteins**		

Kovacs reagent (dimethyl-amino-benzaldehyde)	Roth, Karlsruhe, Germany	Cat#2950.1
Phusion High- Fidelity DNA Polymerase	NEB Biolabs	Cat#M0530S
Immobilon-PSQ Membrane, PVDF, 0,2 μm	Merck, Darmstadt, Germany	Cat#ISEQ00010
DiBAC_4_(3)	Biomol	Cat#ABD-21411
1.6-Diphenyl-1,3,5-hexatriene (DPH)	Thermo Fisher Scientific	Cat#T204

**Critical commercial assays**		

Pierce BCA Protein Assay Kit	Thermo Fisher, Freiburg, Germany	Cat#1074-1395
S-Trap mini columns	PROTIFI, Fairport, USA	Cat#CO2-mini-10
C18-SPE cartridges (25 mg)	Biotage, Uppsala, Sweden	401-055-PX01
sysQUANTOM Viable Cell staining kit	BioCat, Heidelberg	Cat#Q13102-LG

**Deposited data**		

Mass spectrometry data	PRIDE	ProteomeXchange: data set identifier PXD063229

**Plasmids**		

pRS1	Jauss et al.^50^	N.A.
pRS1-YohP _(His)_	this work	N.A.
pRS1 (amp^R^)	this work	N.A.
pRS1-YohP _(Flag)3_	this work	N.A.
pBad24-YohP _(His)_	this work	N.A.
pBad24-YohP _(Flag)3_	this work	N.A.
pKD46	Datsenko & Wanner,^52^	N.A.
pKD3	Datsenko & Wanner,^52^	N.A.
See Table S1 for primers used in this study	this work	N.A.

**Software and algorithms**		

ImageQuant	GE Healthcare	https://www.cytivalifesciences.com/en/us/shop/molecular-biology/nucleic-acid-electrophoresis--blotting--and-detection/molecular-imaging-for-nucleic-acids/imagequant-tl-10-2-analysis-software-p-28619
Image J/Fiji	NIH, Bethesda, USA	https://imagej.nih.gov/ij/index.html
DIA-NN, version 1.8.1	open source	https://github.com/vdemichev/DiaNN
UniProt	Uniprot 2025	https://www.uniprot.org/
MS-DAP-R package	Koopmans et al.^54^	https://github.com/ftwkoopmans/msdap
RStudio	Posit, Boston, USA	https://posit.co/download/rstudio-desktop/
ggplot2	Posit, Boston, USA	https://ggplot2.tidyverse.org/
clusterProfiler	Xu et al.^127^	https://guangchuangyu.github.io/software/clusterProfiler/
STRING	Szklarczyk et al.^64^	https://string-db.org/
org.EcK12.eg.db		https://www.bioconductor.org/packages/release/data/annotation/html/org.EcK12. eg.db.html
LipidXplorer	LipoType GmbH, Dresden	Herzog et al.^128^
Zetasizer software (version 7,13)	Malvern Panalytical, Malvern UK	N.A.
EasyTau Software (version 2.2.3293)	PicoQuant, Berlin	https://www.picoquant.com/products/category/software/easytau-2
GraphPad Prism	Graph Pad Prism Corp., San Diego, USA	https://www.graphpad.com/scientific-software/prism/

### Experimental model and study participant details

#### Strains, growth conditions, and constructs

The *E. coli* K-12 strain MG1655^129^ was used as wild-type strain, and the MG1655 variant containing the chromosomally tagged *yohP* copy (GSO333) was a gift from Dr. Gisela Storz, National Institute of Health, USA. The Δ*relA*,Δ*tnaA*, and Δ*katG* strains were obtained from the KEIO collection^130^ via Thermo Fisher Scientific (Darmstadt, Germany). *E. coli* cells were routinely grown on LB medium at 37°C in either liquid media or on solid media (LB medium + 1.5% agar-agar) unless otherwise stated. The M63 minimal medium was prepared according to Neidhardt et al.^131^

The plasmid pRS1-YohP_(His)_ has been described previously^30^^,^^50^ and encodes a C-terminally His-tagged YohP in the vector pRS1. pRS1 (*amp*^*R*^) contains an pBR322 origin and encodes the *T7* RNA polymerase under the control of the constitutive *EM7* promoter.^50^ pRS1-YohP_(Flag)3_ was constructed via PCR and Gibson Assembly (Protocol E5510 from New England Biolabs) and contained a triple-Flag-tag instead of the His-tag at the C-terminus of YohP. For generating YohP variants in pBad24, the *yohP*-coding sequence including the His_6_ or Flag_3_ sequences of the corresponding pRS1-based plasmids was transferred via Gibson-assembly into pBad24.^51^ All nucleotide primers used in this study are listed in Supplementary Table S3. All PCR reactions were carried out using Q5® High-Fidelity DNA Polymerase (M0491) and Phusion® High-Fidelity DNA Polymerase (NEB Biolabs) according to the manufacturer’s instructions. The Gibson Assembly® Protocol from New England Biolabs was used for incorporating DNA sequences into the plasmids used in this study – according to the manufacturer’s instructions. Production of YohP from either pRS1-YohP_(His)_ or pBad-YohP_(His)_ was usually induced at OD 0.2 - 0.4 with either 1 mM IPTG or 0.2% arabinose for approx. 2 - 4 h.

For generating the Δ*yohP* strain, the *E. coli* K12 MG1655 strain was transformed with the temperature-sensitive pKD46 plasmid^52^ and cells were grown at 30 °C. The chloramphenicol acetyltransferase gene flanked by FRT sites was amplified from plasmid pKD3. Cells with the pKD46 plasmid were grown in LB medium at 30 °C and induced with 10 mM L-arabinose after OD_600_ reached approximately 0,1 to induce expression of the λ-red genes. The growth was continued at 30 °C until OD_600_=0,4. Then aliquots of 1ml were taken into two 1,5 mL centrifuge tubes and kept in ice for 10 minutes, centrifuged for 10 minutes at 4000 x g and 4 °C, after which the pellet was resuspended in 1 ml of ice-cold sterile H_2_O. The linear PCR fragment containing the chloramphenicol acetyltransferase gene flanked by FRT sites and sequences adjacent to the *yohP* gene was then electroporated into the MG1655 pKD46 strain, followed by incubation for 2 h with moderate shaking at 30 °C. Aliquots were plated on Cm-containing plates, and Δ*yohP* colonies were validated via colony PCR.

Permission to work with genetically modified organisms was granted by the Regierungspräsidium Tübingen (Uni.FR.27.02/18.01).

### Method details

#### Native YohP production under stress conditions

Cell envelope stress was induced by the addition of 1 mM EDTA and 0.025% SDS at OD_600_ = 0.2 and cultures were further incubated up to OD_600_ = 1.5. H_2_O_2_ (1 mM) was added to LB-grown cultures at OD_600_ = 0.2 to induce oxidative stress and cells were further incubated up to OD_600_ = 1.5. Oxygen-limited cultures were inoculated from LB-grown overnight cultures into 2 ml LB medium supplemented with 0.2% deoxygenated glucose in 2-ml Eppendorf tubes and cells were grown without shaking. The corresponding aerobic control cultures were inoculated from the same overnight cultures into 5 ml LB plus 0.2% glucose in 50-ml Falcon tubes and were grown with shaking. Cells from both sets of samples were harvested at an optical density at 600 nm (OD_600_) of ∼1.5.

#### SDS-PAGE, immune detection and antibodies

For immune detection, proteins were separated via denaturing SDS-polyacrylamide gel electrophoresis (SDS-PAGE). Two different gel systems were used in this study: 15% Tris-Glycine PAGE for the separation of proteins between 10-100 kDa and 16.5% Tris-Tricine PAGE for the separation of small proteins between 0,5-10 kDa.^30^ Samples were denatured at 37°C or 56°C for 10 min in Laemmli loading buffer (278 mM Tris-HCl, pH 6.8, 44.4% glycerol, 4.4% SDS, 0.02% bromophenol blue) containing fresh DTT at a final concentration of 25 mM. For the immune detection of proteins in whole cells, routinely 1 x 10^8^ cells were precipitated with 5 % trichloroacetic acid (TCA, final concentration) and incubated for 30 min on ice. The sample was subsequently centrifuged at 30.000 x g and 4 °C for 15 min, the pellet was resuspended in 20 μl of SDS loading buffer and incubated for 10 min at 56 °C before loading on SDS-PAGE. The SDS-PAGE-separated proteins were then transferred via tank blot onto 0.45 μm nitrocellulose membranes (GE Healthcare, Freiburg, Germany) at 750 mA for 2 hours. For the detection of YohP, the gel was transferred by semi-dry western blotting onto 0,22μm PVDF PsQ membranes (Merck, Darmstadt, Germany). Membranes were blocked with 5% milk powder in T-TBS buffer for at least 1 h before the addition of the primary antibodies.

α-Flag antibodies (mouse α-Flag M2) were obtained from Merck (Darmstadt, Germany). PyrD antibodies (rabbit α-PyrD, CSB-PA812570XA01FQR) and PspC antibodies (rabbit α-PspC, CSB-PA365328XA01SZB) were obtained from Cusabio via Biozol (Hamburg, Germany). PspA antibodies were a kind gift from Dirk Schneider, Univ. Mainz. Peptide antibodies against RpoS have been described before.^132^ Antibodies against YidC were raised against the purified proteins and have been reported before.^125^^,^^126^ Polyclonal TnaA antibodies were obtained from AssayPro (No. 33517-05111; St. Charles, USA), and α-His antibodies were from Invitrogen (MA1-21315). Horseradish peroxidase-coupled goat α-rabbit and goat α-mouse secondary antibodies were obtained from SeraCare (Milford, MA, USA). Blots were incubated for 1 min with home-made ECL reagent and signals were detected by a CCD camera. Western blot samples were analyzed by using *ImageQuant* (GE Healthcare) or the *ImageJ/ Fiji* plug-in software (NIH, Bethesda, USA). All western blot experiments were performed at least twice as independent biological replicates and representative gels/blots/images are shown. When data were quantified, at least three independent biological replicates with several technical replicates were performed, and the signal intensity observed for wild-type cells or cell extracts was set to 100%.

#### Mass spectrometry of *E. coli* cells

For determining differentially produced proteins in response to *yohP*-deletion or expression, proteomic analyses were performed. *E. coli* MG1655, *E. coli* MG1655 pRS1-YohP_(His)_ plasmid and *E. coli* MG1655 Δ*yohP* were grown on phosphate-rich medium (INV medium; 10 g yeast extract, 10 g tryptone/peptone, 1% glucose, 1mM KH_2_PO_4_, 166 mM K_2_HPO_4_) YohP-producing cells were induced at the OD_600_ = 0.5 with 1mM IPTG and grown for additional 2h. All samples were harvested at the OD_600_ = 1.5-1.7 and subjected to cell lysis via 3 cycles through the Emulsiflex cell lysis system at 800 psi. The homogenate was centrifuged at 15.000 rpm in an SS34 rotor (Beckmann-Coulter, Krefeld, Germany) and the protein concentrations in the supernatants were determined via the Pierce™ BCA Protein Assay Kit (Thermo Fisher, Freiburg, Germany).

A volume of cell lysate containing 200 μg of proteins was mixed 1:1 with SDS buffer (10% SDS, 100 mM tetraethyl ammonium bicarbonate (TEAB) pH 8.5) and subjected to tryptic digestion following the S-Trap protocol^133^ with small modifications. Briefly, proteins were reduced by the addition of 4 μL TCEP for 30 min at 37 °C with mild agitation, and alkylated with 8 μL iodoacetamide (IAA) for 30 min in the dark with mild agitation. Samples were acidified with 12 μL 12% phosphoric acid, diluted with 750 μL of the S-Trap binding buffer (1 M TEAB Buffer and Methanol (10:90)) and transferred to S-Trap mini columns (PROTIFI, Fairport, USA). Three washing cycles with 400 μl S-Trap binding buffer were done prior to digestion for SDS removal. MS-grade Trypsin (Serva, Heidelberg, Germany) was added in an enzyme-to-protein ratio of 1:50 using 125 μL digestion buffer (50mM ammonium bicarbonate (ABC), pH 8.5) as media and the column was incubated overnight at 37 °C with light agitation. Peptides were eluted stepwise in 80 μL 50mM ABC, 0.2% formic acid, 50% acetonitrile (ACN). The pooled fractions were diluted 1:1 with 0.1% trifluoroacetic acid (TFA) and desalted with C18-SPE cartridges (25 mg, Biotage, Uppsala, Sweden). After equilibration with 2 mL ACN, 1 mL 50% ACN/1% acetic acid, and 2 mL 0.1% TFA the samples were loaded onto the cartridge, washed with 2 mL 0.1% TFA and eluted with 1 mL 80% ACN/0.1% TFA. The eluted fractions were dried using an Eppendorf concentrator (Eppendorf) and stored at -20 °C.

Dried peptides were reconstituted in 5% ACN with 0.1% formic acid (FA). Peptides were loaded onto an Acclaim PepMap C18 capillary trapping column (particle size 3 μm, L = 20 mm) and separated on a ReproSil C18-PepSep analytical column (particle size = 1.9 μm, ID = 75 μm, L = 50 cm, Bruker Corporation, Billerica, USA) using a nano-HPLC (Dionex U3000 RSLCnano) at a temperature of 55 °C. Trapping was carried out for 6 min with a flow rate of 6 μL/min using a loading buffer composed of 0.05% trifluoroacetic acid in H_2_O. Peptides were separated by a gradient of water (buffer A: 100% H_2_O and 0.1% FA) and acetonitrile (buffer B: 80% ACN, 20% H_2_O, and 0.1% FA) with a constant flow rate of 250 nL/min. The gradient went from 4% to 458% buffer B in 9120 min. All solvents were LC-MS grade and purchased from Riedel-de Häen/Honeywell (Seelze, Germany). Eluting peptides were analyzed in data-independent acquisition mode on an Orbitrap Eclipse mass spectrometer (Thermo Fisher Scientific) coupled to the nano-HPLC by a Nano Flex ESI source.

MS1 survey scans were acquired over a scan range of 350 – 1650 m/z in the Orbitrap detector (resolution = 120k, automatic gain control (AGC) = 5e^5^, and maximum injection time = 100 ms). Sequence information was acquired by a tMS2 method with the following settings. MS2 scans were generated in a scan range of 350 – 2000 m/z with isolation windows proposed by Muntel et al.^134^ Fragmentation was induced by higher-energy collisional dissociation (HCD) at 27% normalized collision energy (NCE). For Orbitrap MS2, an automatic gain control (AGC) of 1xe^6^ with dynamic injection time and a minimum of 6 desired points across the peak (resolution = 30k).

MS raw files were processed with the open-source software DIA-NN (version 1.8.1)^135^ using a library-free approach. The predicted library was generated by in silico digesting the *Escherichia coli* UniProt reference proteome (UP000000625, 4399 entries, downloaded in March 2023) with Trypsin/P. Deep learning-based spectra- and RT-prediction were enabled. Peptide length range was set to 6 - 35 amino acids, missed cleavages to 2 and precursor charges to 1-5. Methionine oxidation and N-terminus acetylation were set as variable modifications, and cysteine carbamidomethylation as a fixed modification. The maximum number of variable modifications per peptide was limited to 1. MS1 and MS2 accuracies were automatically calculated by DIA-NN. Protein inference was performed using genes with the heuristic protein inference option enabled. The neural network classifier was set to single-pass mode, and the quantification strategy was selected as ‘QuantUMS (high precision)’. Reported data from DIA-NN were analyzed using the MS-DAP-R package^54^ with the following parameters: filter minimum detect = 2, filter minimum quant = 2, filter minimum peptide per protein = 1, filter by contrast = true, normalization algorithm = vsn followed by mode between_protein, differential expression analysis algorithm = deqms, and q-Value threshold = 1%.

#### Downstream analysis of proteomics data

Outputs from the differential expression analysis were imported in RStudio for downstream analysis, and all plots were generated using ggplot2. For all downstream analysis, proteins with a statistically significant differential expression (q-value < 0.05) and with a log2-fold change of at least |1| were selected.

GO enrichment analysis was performed using clusterProfiler^127^ with the org.EcK12.eg.db as source for annotations. The obtained p-values in the GO analysis were corrected using the Benjamin-Hochberg method with a 5% confidence level. GO analysis was performed separately for up- and downregulated proteins, and for GO annotations linked to biological processes and cell compartment.

Lists of differentially expressed proteins were also uploaded to STRING^64^ for network analysis. Interaction networks were generated for up- and downregulated proteins separately and combined. Non-interacting nodes were removed from the final output, and a minimum interaction score of 0.4 was used.

#### Indole measurements

*E. coli* cells for indole measurements were grown on INV medium or tryptone broth (10g/L tryptone, 5g NaCl/L) up to the indicated optical density. Cells containing pRS1-YohP_(His)_ were induced with 1 mM IPTG. After centrifugation at 20.000 g for 30 min at 4 °C, the indole concentration in the supernatant was determined using the Kovacs reagent (dimethyl-amino-benzaldehyde) (Roth, Karlsruhe, Germany).^136^ In brief, 10 μl of the supernatant were incubated with 200 μl Kovacs reagent in a 96-well plate for 1 h at room temperature. The formed cyan-dye rosindole was determined by measuring the absorption at 571 nm with a Tecan Spark plate reader, using an indole standard curve (0-1 mM indole) as reference.

#### Isolation of inner membrane vesicles for lipidomics

Lipid composition was determined in inner membrane vesicles (INVs) of the indicated strains. Cells containing pRS1-YohP_(His)_ were induced with 1 mM IPTG. Cells were grown on INV medium up to OD_600_ of 1.5, harvested, and washed twice in buffer A (50 mM TeaOAc, pH 7.5; 250 mM sucrose, 1mM EDTA, 1 mM DTT). Cells were resuspended in 1 ml buffer A/g cell weight and lysed in a cooled French pressure cell. The lysate was then centrifuged at 150.000 g for 2 h in a Beckmann Ti50.2 rotor, and the pellet containing the membranes was resuspended in buffer A. These crude membranes were loaded on a discontinuous sucrose gradient (0.77 M; 1.44 M; 2.02 M sucrose in buffer A + 0.5 mM PMSF) and centrifuged in a Sorvall SureSpin106.4 swing-out rotor at 25.000 rpm for 17h. After centrifugation, the inner membrane phase was collected, diluted with 50 mM TeaOAc, pH 7.5 and centrifuged for 2 h at 150.000 g in a Beckmann TI50.2 rotor. The pellet was resuspended in buffer A, and samples were stored in small aliquots at -80 °C.

#### Lipid extraction for lipidomics

Mass spectrometry-based lipid analysis was performed by Lipotype GmbH (Dresden, Germany) as described.^137^ Lipids were extracted using a two-step chloroform/methanol procedure.^138^ Samples were spiked with internal lipid standard mixture containing: cardiolipin 14:0/14:0/14:0/14:0 (CL), ceramide 18:1;2/17:0 (Cer), diacylglycerol 17:0/17:0 (DAG), hexosylceramide 18:1;2/12:0 (HexCer), lyso-phosphatidate 17:0 (LPA), lyso-phosphatidylcholine 12:0 (LPC), lyso-phosphatidylethanolamine 17:1 (LPE), lyso-phosphatidylglycerol 17:1 (LPG), lyso-phosphatidylinositol 17:1 (LPI), lyso-phosphatidylserine 17:1 (LPS), phosphatidate 17:0/17:0 (PA), phosphatidylcholine 17:0/17:0 (PC), phosphatidylethanolamine 17:0/17:0 (PE), phosphatidylglycerol 17:0/17:0 (PG), phosphatidylinositol 16:0/16:0 (PI), phosphatidylserine 17:0/17:0 (PS), cholesterolester 20:0 (CE), sphingomyelin 18:1;2/12:0;0 (SM), triacylglycerol 17:0/17:0/17:0 (TAG). After extraction, the organic phase was transferred to an infusion plate and dried in a speed vacuum concentrator. First step dry extract was resuspended in 7.5 mM ammonium acetate in chloroform/methanol/propanol (1:2:4, V:V:V), and the second step dry extract in 33% ethanol solution of methylamine in chloroform/methanol (0.003:5:1; V:V:V). All liquid handling steps were performed using Hamilton Robotics STARlet robotic platform with the Anti-Droplet Control feature for organic solvents pipetting.

#### Lipidomics data acquisition and analyses

Samples were analyzed by direct infusion on a QExactive mass spectrometer (Thermo Scientific) equipped with a TriVersa NanoMate ion source (Advion Biosciences). Samples were analyzed in both positive and negative ion modes with a resolution of Rm/z=200=280000 for MS and Rm/z=200=17500 for MSMS experiments, in a single acquisition. MSMS was triggered by an inclusion list encompassing corresponding MS mass ranges scanned in 1 Da increments.^139^ Both MS and MSMS data were combined to monitor CE, DAG, and TAG ions as ammonium adducts; PC, PC O-, as acetate adducts; and CL, PA, PE, PE O-, PG, PI, and PS as deprotonated anions. MS only was used to monitor LPA, LPE, LPE O-, LPI, and LPS as deprotonated anions; Cer, HexCer, SM, LPC and LPC O- as acetate adducts.

Data were analyzed with in-house developed lipid identification software based on LipidXplorer.^128^ Data post-processing and normalization were performed using an in-house developed data management system. Only lipid identifications with a signal-to-noise ratio >5, and a signal intensity 5-fold higher than in corresponding blank samples were considered for further data analysis.

#### Time-resolved DPH anisotropy analysis of INV

INVs were prepared as described above. and lipid concentrations of the INV dispersions were determined by the Bartlett assay.^140^ The homogeneity of the INV samples was validated by dynamic light scattering (DLS) measurements, which were conducted at 20°C using a Nano-ZS Zetasizer from Malvern Panalytical (Kassel, Germany). The instrument was equipped with a 633 nm He-Ne laser and detected at an angle of 173°. Data were collected and analyzed using Zetasizer software (version 7,13), which also calculated the viscosity and refractive index of the medium from its database. The software automatically optimized the attenuator and measurement position and was also used to determine particle size and size distribution. The INV samples were adjusted to a total protein concentration of 1 mg and 0.1 mol% DPH (< 1 vol% DMSO) along with the buffer solution (10 mM Tris, 100 mM NaCl, 0.02% NaN3, pH 7,4 at 20°C) were added. The samples were incubated for 10 minutes at 20°C with stirring at 400 rpm. Time-resolved anisotropy measurements were performed at 20 °C using a FluoTime 300 spectrometer from PicoQuant (Berlin, Germany) with 100 μL volume Ultra-Micro cells from Hellma (Mulheim, Germany) having an optical path of 10 x 2 mm. The setup and initial data analysis were conducted using EasyTau Software (version 2.2.3293). Excitation was done at 355 nm with a laser polarization of 0° at a frequency of 16,67 MHz, laser intensity of 7,2, and a pulse width of 25 ps. Emission was measured through a 355 nm long-pass filter at 430 nm with a 5 nm detection band pass, and the emission polarizer set to 0°, 54.7°, and 90°, respectively. The G-factor was recorded with the same setup with laser polarization at 90° and calculated by manually aligning emission decays at polarizations of 0° and 90°. The anisotropy decay was obtained by a tail fit of the decay curves with vertical and horizontal polarizers and fitted assuming a mono-exponential decay in terms of the initial anisotropy, the correlation time of the angular motion of DPH, and the limiting anisotropy, r_∞_, extrapolated to infinite time after excitation. The latter parameter represents the angular constraints to the tumbling of the probe, so that a large r_∞_ stands for relatively high order and tight packing of the acyl chains. The goodness of fit was assessed using reduced chi-square values and bootstrap error analysis, although these are not shown. The time-resolved DPH anisotropy analysis was performed using three independent biological replicates of INVs, each containing three technical replicates.

#### Membrane potential measurement in whole cells

The *E. coli* K12 MG1655 strain with the indicated plasmids were grown on LB and protein production was induced at OD_600_ = 0.5 and further incubated at 37 °C for 2 h. Aliquots of 1x10^9^ cells were collected from each sample and washed once with sterile PBS. When indicated 1mM of CCCP (100mM stock in DMSO) was added. All samples were adjusted to a total volume of 100 μl and further incubated for 30 minutes at 37 °C. The samples were washed with PBS and diluted with 500 μl of PBS supplemented with 2μg final concentration of the dye DiBAC_4_(3) (Stock solution: 10 mg/ml in DMSO, working solution: 200 μg/ml in sterile deionized H_2_O, freshly prepared). Samples were incubated at room temperature for 20 minutes in the dark, centrifuged at 13.000 rpm for 3 min at room temperature, washed 2x with sterile PBS, and incubated with 2% paraformaldehyde in a total volume of 100 μl PBS for 10 min at room temperature. Samples were then washed with PBS and finally resuspended in 200 μl PBS. 100 μl from this solution was transferred to a black 96-well plate (Greiner, Frickenhausen, Germany) and fluorescence was measured in a Tecan Spark plate reader after excitation at 485 nm and an emission of 510 nm.

#### *In vivo* metabolic labeling

*E .coli* MG1655 containing either plasmid pBad24 or pBad-YohP_(His)_ were grown and induced for 2 h on M63 minimal medium^131^ supplemented with 20 amino acids. Cells were harvested and washed 3 times with M63 medium lacking methionine and cysteine, resuspended in the same medium and induced for an additional 30 minutes. An aliquot of 2x10^8^ cells of each sample was then added to 1ml M63 medium and supplemented with 1 μCi ^35^S-methionine and cysteine labeling mix (Perkin Elmer, Rodgau, Germany). 100 μl samples were taken from each culture at different time points after the addition of radioactive labeling mix and directly precipitated with 5% TCA, separated with SDS PAGE and visualized using autoradiography.

#### Viable cell staining and counting

The assay was performed using the QUANTOM Tx Microbial Cell Counter and the QUANTOM™ Viable Cell Staining Kit obtained from BioCat GmbH (Heidelberg, Germany). The kit stains live bacterial cells to be counted. The optical density of the cell culture was determined and an aliquot corresponding to approx. 1 x 10^8^ cells were collected. The cells were washed with PBS and the culture media was completely removed. Cells were resuspended in PBS buffer and incubated with different CCCP concentrations or DMSO as a control for 1 h at 37 °C. Subsequently, the cells were collected by centrifugation and washed with QUANTOM Cell Dilution Buffer, centrifuged and resuspended in 80 μL QUANTOM Cell Dilution Buffer. 10 μL of this suspension from each sample were transferred into a fresh tube and incubated with 2 μL of the QUANTOM™ Viable Cell Staining Dye was added and mixed gently and carefully. The cells were then incubated at 37°C for 30 minutes in the dark. Thereafter 8.0μL QUANTOM™ Cell Loading Buffer I was added and mixed gently without creating bubbles. 5.0μL of this mixture was loaded onto a QUANTOM™ M50 Cell Counting Slide and centrifuged at 300 x g for 10 minutes in a QUANTOM™ Centrifuge at room temperature. The slide was then inserted into the QUANTOM Tx™ cell counter and cells were counted with the light intensity level set to either 7 or 9. The obtained viable cell numbers per ml were then plotted against the CCCP concentration.

For monitoring the survival rate of *E. coli* cells in the presence of the antibiotic ciprofloxacin, cells were treated for 6 h at 37 °C with 5 μg/ml ciprofloxacin during exponential phase (OD_600_ ∼0.4).^40^ The survival rate in the presence of H_2_O_2_ was determined by treating the cells for 30 min with 2 mM H_2_O_2_.^141^ Pre- and post-treatment samples were used to determine the relative survival rate.

#### (p)ppGpp analyses by CE-MS

The concentration of (p)ppGpp was determined by capillary electrophoresis-mass spectrometry (CE-MS). *E. coli* cells were grown on LB medium up to OD_600_ = 0.8 and 20 x 10^8^ cells were lysed with pre-chilled formic acid (final concentration 1 M) and stored at -80 °C until further extraction. Samples were thawed and spiked with heavy [^15^N]_5_ (p)ppGpp standards before extraction. Following incubation for 30 minutes with vortexing every two minutes, samples were diluted with 50 mM NH_4_OAc, pH 5.5, and centrifuged (10 min, 3220 g, 4 °C). The supernatant was subjected to weak anion solid phase extraction using a GX-241 ASPEC system (Gilson Inc, Middelton, USA) and EVOLUTE® EXPRESS WAX cartridges (100 mg/3 ml (Tabless)) from biotage (Uppsala, Sweden), which were equilibrated with MeOH (1ml) and NH_4_OAc (1 ml, 50 mm, pH 4.5) before samples were loaded onto the column. Cartridges and analytes were washed with NH_4_OAc (1 ml, 50 mm, pH 4.5) and MeOH (1 ml). Samples were eluted using a MeOH:ddH_2_O:NH_4_OH buffer (20:70:10, 2 × 750 μl), diluted with ddH_2_O (2 ml) and lyophilized overnight. The precipitate was dissolved in ddH_2_O (100 μl), and the solvent was removed using an Eppendorf vacuum concentrator for 5h at room temperature. The obtained precipitate was dissolved in 60 μl ddH_2_O. The extracted samples were stored at -20 °C until measurement. Sample preparation for the CE-MS measurement was performed by centrifugation of the samples and further dilution of the aqueous sample in a ratio of 1:1 with ddH_2_O.

Capillary electrophoresis (CE) was performed using an Agilent 7100 CE system (Agilent Technologies, Waldbronn, Germany). The CE was coupled to an Agilent G6495C Agilent QQQ mass spectrometer via a commercial Agilent jet stream (AJS) electrospray ionization (ESI) source and an Agilent liquid coaxial interface. The analyte solution was diluted with a ddH_2_O-isopropanol mixture (1:1 v-%), using an Agilent 1200 isocratic LC pump and a 1:100 splitter. The resulting flow was 10 μl/min. A bare fused silica capillary (100 cm length, 50 μm internal diameter) was activated and flushed with 1 M NaOH and water for 10 minutes before the first measurement. At the beginning of each measurement, the capillary was washed with ddH_2_O (300 s) and ammonium acetate serving as separation buffer (35 mm, pH 9.75, 300 s). After sample injection with a pressure of 100 mbar for 10 s and a buffer plug (50 mbar, 5 s), a voltage of +30 kV was applied for separation, which resulted in a stable current of 22 μA. The MS ran in negative ionization mode with a capillary voltage of -2000 V and a nozzle voltage of 2000 V. The pressure RF was set to an upper limit of 90 V and a lower limit of 60 V. Nebulizer gas was set to 8 psi with a temperature of 150 °C and a flow of 11 l/min. The sheath gas had a temperature of 175 °C and a flow of 8 l/min. Peak assignment was performed with internal heavy standards and MS/MS transitions.

### Quantification and statistical analysis

Western blot and autoradiography samples were analyzed by using the *ImageQuant* (GE Healthcare) or the *ImageJ/Fiji* plug-in software (NIH, Bethesda, USA). All experiments were performed at least twice as independent biological replicates, and representative gels/blots/images are shown. When data were quantified, at least three independent biological replicates with several technical replicates were performed. Mean values and standard deviations were determined by using either Excel (Microsoft Corp.) or GraphPad Prism (GraphPad Prism Corp., San Diego). To determine the significance of the results, the P-value was calculated using the `unpaired, two-tailed t-test' of the program Graph Pad PRISM 6 or the one-way Anova analyses and Turkey’s honest significance test using the values in the wild-type strain as reference.

## Supplemental information

Document S1. Figures S1–S7 and Table S3

Table S1. Differentially expressed proteins in the *ΔyohP* strain (Table S1.exe), related to Figure 3

Table S2. Differentially expressed proteins in the *yohP*-expressing strain (Table S2.exe), related to Figure 3

Data S1. Differentially expressed proteins in the Δ*yohP* strain

Data S2. Differentially expressed proteins in the *yohP*-expressing strain
