# Supplementary material for: Metabolic silencing induced by the small bacterial membrane protein YohP
Source: iScience. 2025 Nov 19;28(12):114123. doi: 10.1016/j.isci.2025.114123 (PMC12719787; doi:10.1016/j.isci.2025.114123)
Supplement: Document S1. Figures S1–S7 and Table S3 [file mmc1.pdf]

iScience, Volume *28*

Supplemental information

Metabolic silencing induced by the small bacterial membrane protein YohP

Ana Natriashvili, Nahid Mohammadsadeghi, Martin Milanov, Eva Smudde, Isabel Prucker, Henning J. Jessen, Iulia Carabadjac, Heiko Heerklotz, Pedro H.C. Franco, Julian D. Langer, and Hans-Georg Koch

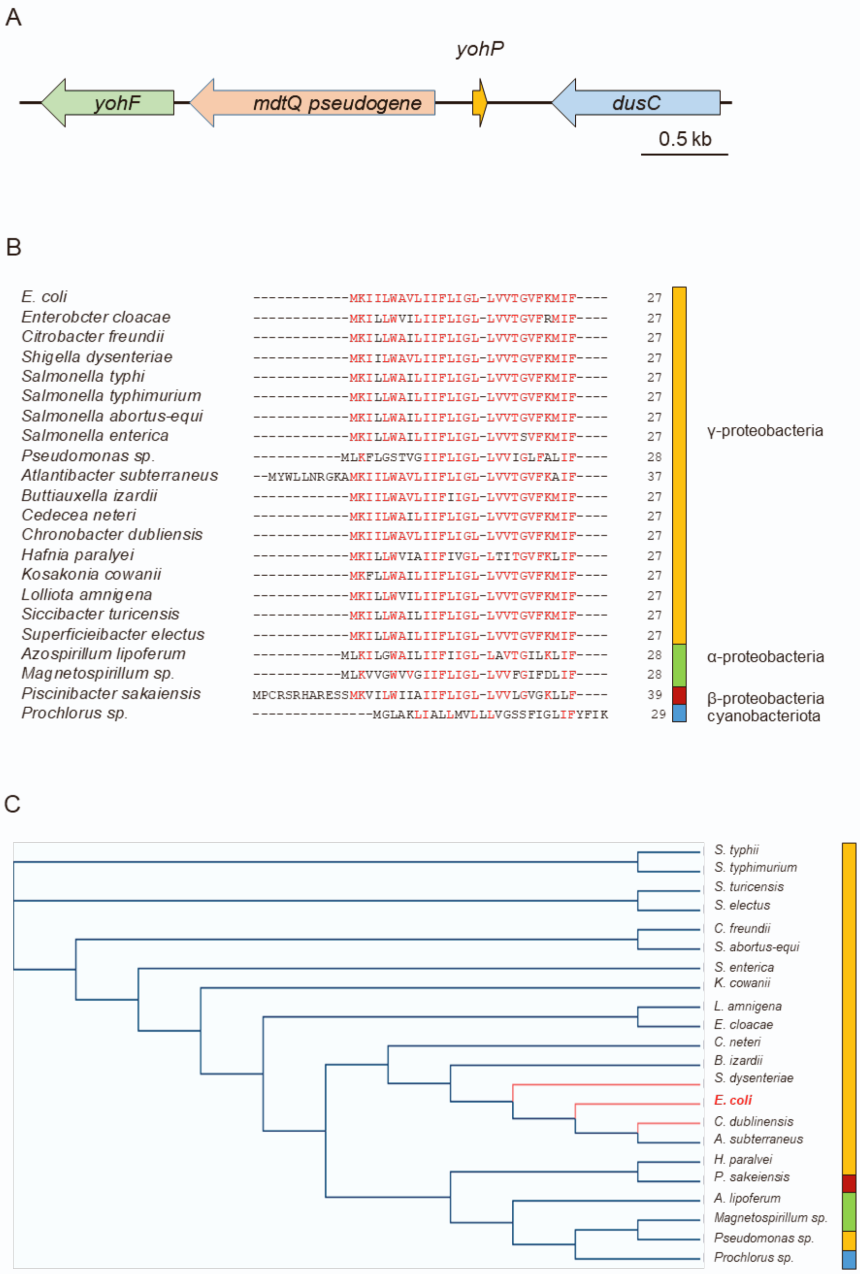

Figure S1. Genomic context and sequence conservation of bacterial YohP homologues (Related to introduction). **A.** Genomic context of *E. coli* YohP. *yohF* encodes for a putative oxidoreductase, *mdtQ* for a pseudogene with homology to multidrug resistance outer membrane proteins. Note that although *mdtQ* is classified as pseudogene in *E. coli* MG1655, other *E. coli* strains appear to have intact alleles. *DusC* encodes for a tRNA-dihydrouridine16 synthase. The genomic context is conserved in many proteobacterial species. **B.** Sequence alignment of YohP from different bacterial species. YohP homologues are mainly present in γ-proteobacteria, but are also found in some species outside of this group. The sequences were retrieved from UniProt and alignments were performed with Clustal 1.2.4. and the NCBI Constraint-based Multiple Alignment Tool (COBALT). **C.** Neighbor-joining tree for the YohP homologues shown in B. The tree was calculated without distance correction and at a maximum sequence difference of 0.8. The tree was generated using the NCBI Tree viewer based on the COBALT sequence alignment.

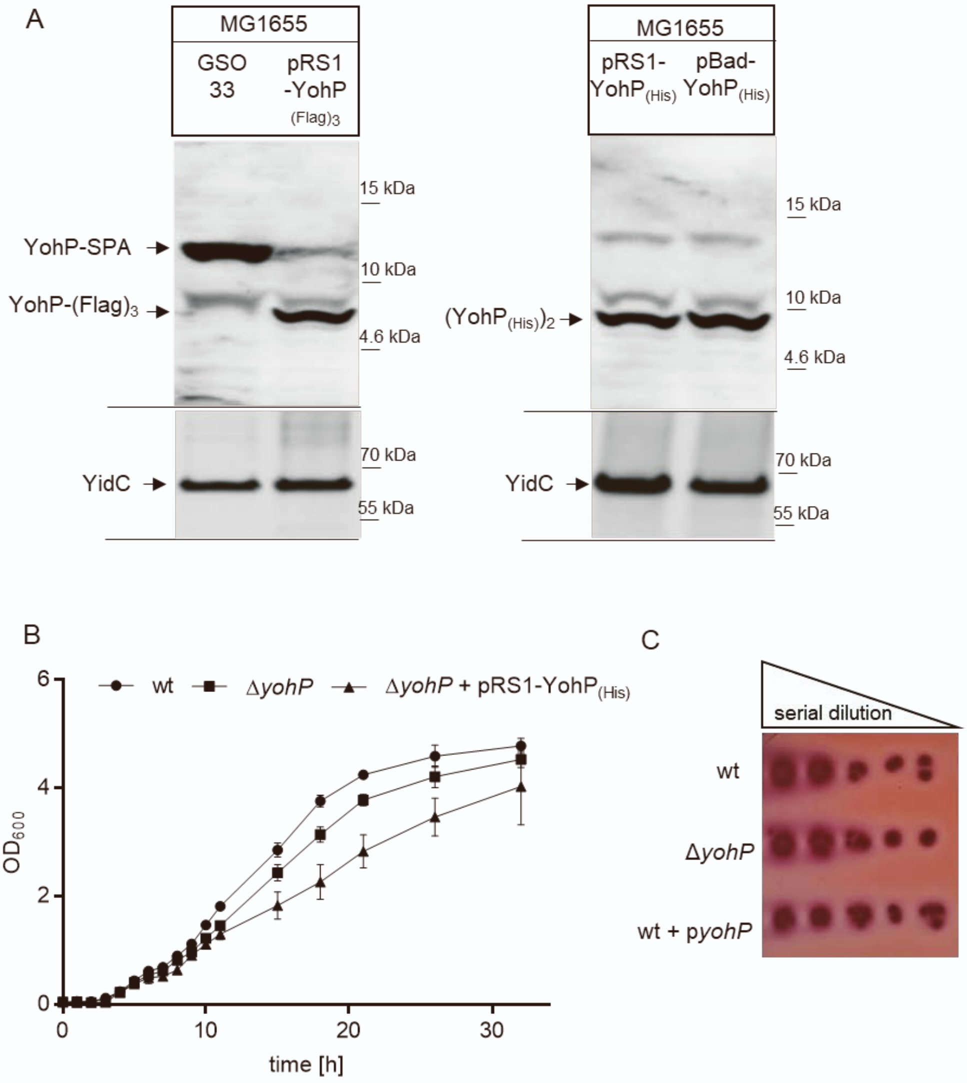

Figure S2. The YohP levels are not influenced by the affinity tag (Related to Figs 1 & 2). **A.** The indicated *E. coli* cells were grown on LB medium up to an optical density of 1.5. Expression of yohP from the plasmids was induced at OD_{600} = 0.4 with 1 mM IPTG or 0.2% arabinose for 2-4 h. 2 x 10^8 cells were precipitated with 5% trichloroacetic acid (TCA). After centrifugation, samples were denatured and separated on 16.5 % Tris-Tricine SDS-PAGE, followed by western transfer. The membrane was then cut and one part was incubated with α-Flag antibodies (left panel) and the other one with α-His antibodies (right panel). The upper parts of both membranes were decorated with α-YidC antibodies. Note, that the SPA- or triple-Flag-tagged YohP variants do not form SDS-resistant dimers and are detected as monomers. In contrast, His-tagged (and untagged; not shown) YohP versions are present as dimers. **B.** The indicated MG1655 *E. coli* strains were grown on M63 minimal medium and growth was monitored via optical density readings. The medium for the plasmid-containing strain contained 1 mM IPTG. Precultures of the indicated strains were grown on LB medium **C.** Precultures of the indicated strains were grown on LB medium up to OD_{600}=0.8, washed and serially diluted with PBS buffer and spotted on MacConkey agar with maltose as single carbon source.

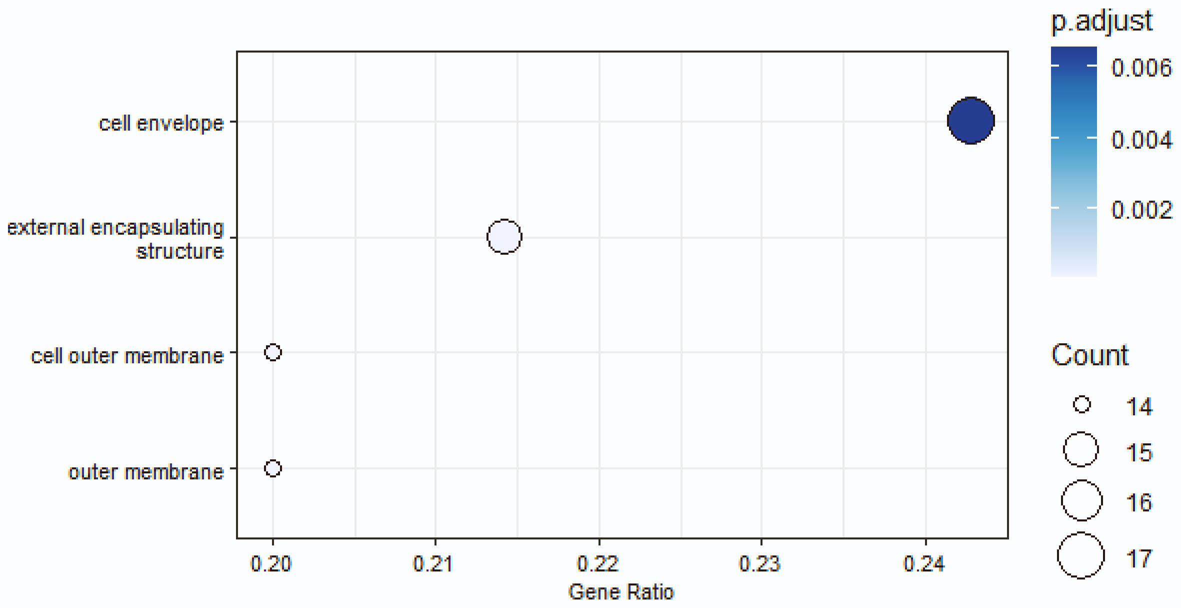

Figure S3: Upregulated proteins upon *yohP*-induction are localized to the cell envelope and outer membrane (Related to Fig. 3). Dotplots for the GO classification of Cellular Component (CC) enrichment analysis for the comparison between wild-type *E. coli* and the *yohP*-induction strain.

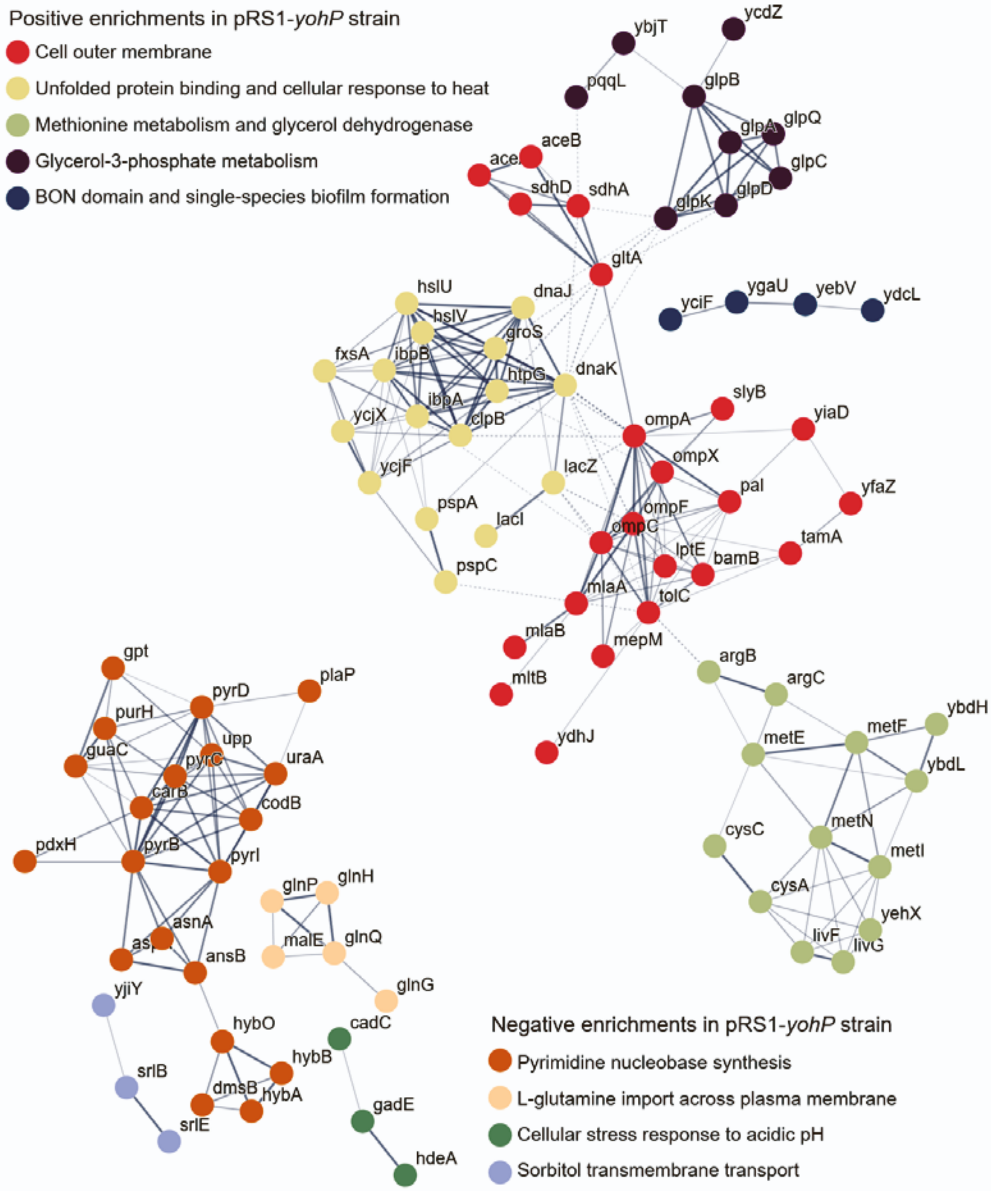

Figure S4: YohP-producing cells contain well-connected groups of differentially expressed proteins (Related to Fig. 3). String networks for the comparison between wild-type *E. coli* and the *yohP*-induction strain. Only differentially abundant proteins (q-value < 0.05 and fold-change > 2) were selected for the network analysis, and non-interacting nodes were removed. K-means clustering analysis of the network led to significant nodes (p < 0.05) grouped by different colors.

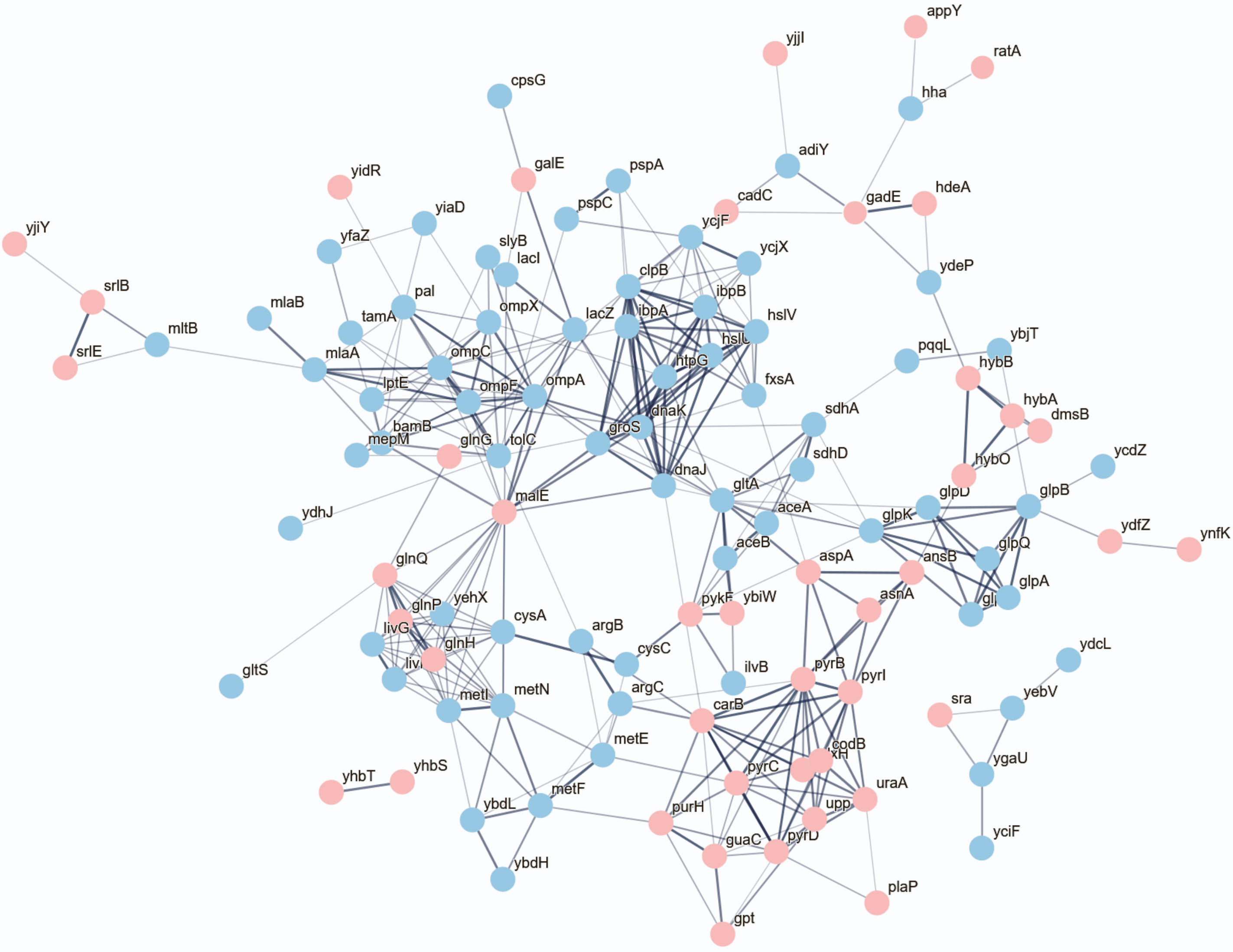

Fig. S5: STRING network analyses of up- and down-regulated proteins upon YohP production (Related to Fig. 3). STRING analyses were performed as in Fig. S4 and up-regulated proteins are labeled in blue, while down-regulated proteins are labeled in red.

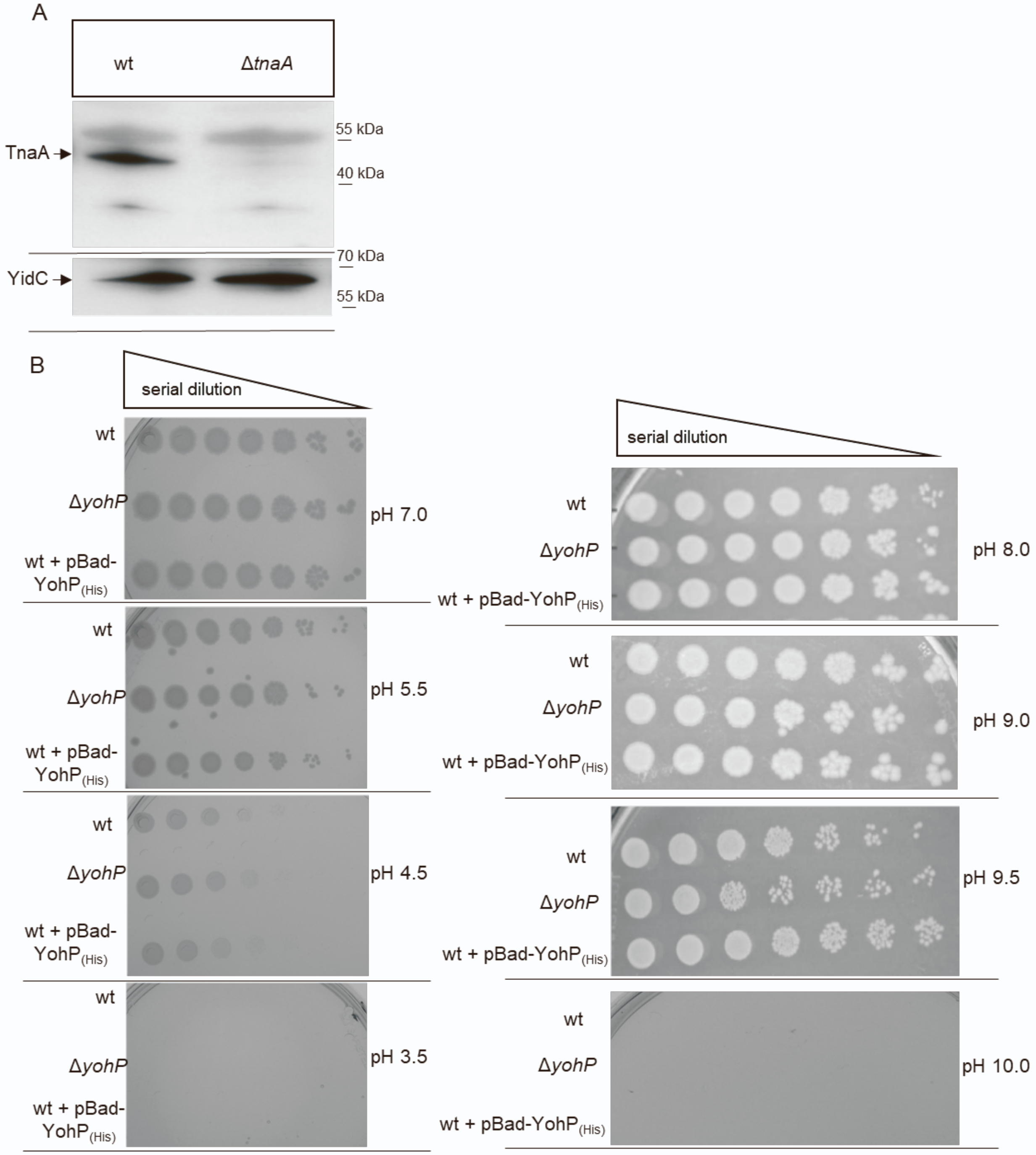

Fig. S6: Acid resistance is not influenced by the YohP levels (Related to Fig. 4). **A**. The indicated strains were grown on LB medium and processed as described in the legend to Fig. S2. Immune detection using α-TnaA antibodies confirmed the specificity of the antibody. **B.** The indicated strains were grown overnight on LB-medium, washed and serially diluted with PBS buffer and spotted onto agar plates with different pH values.

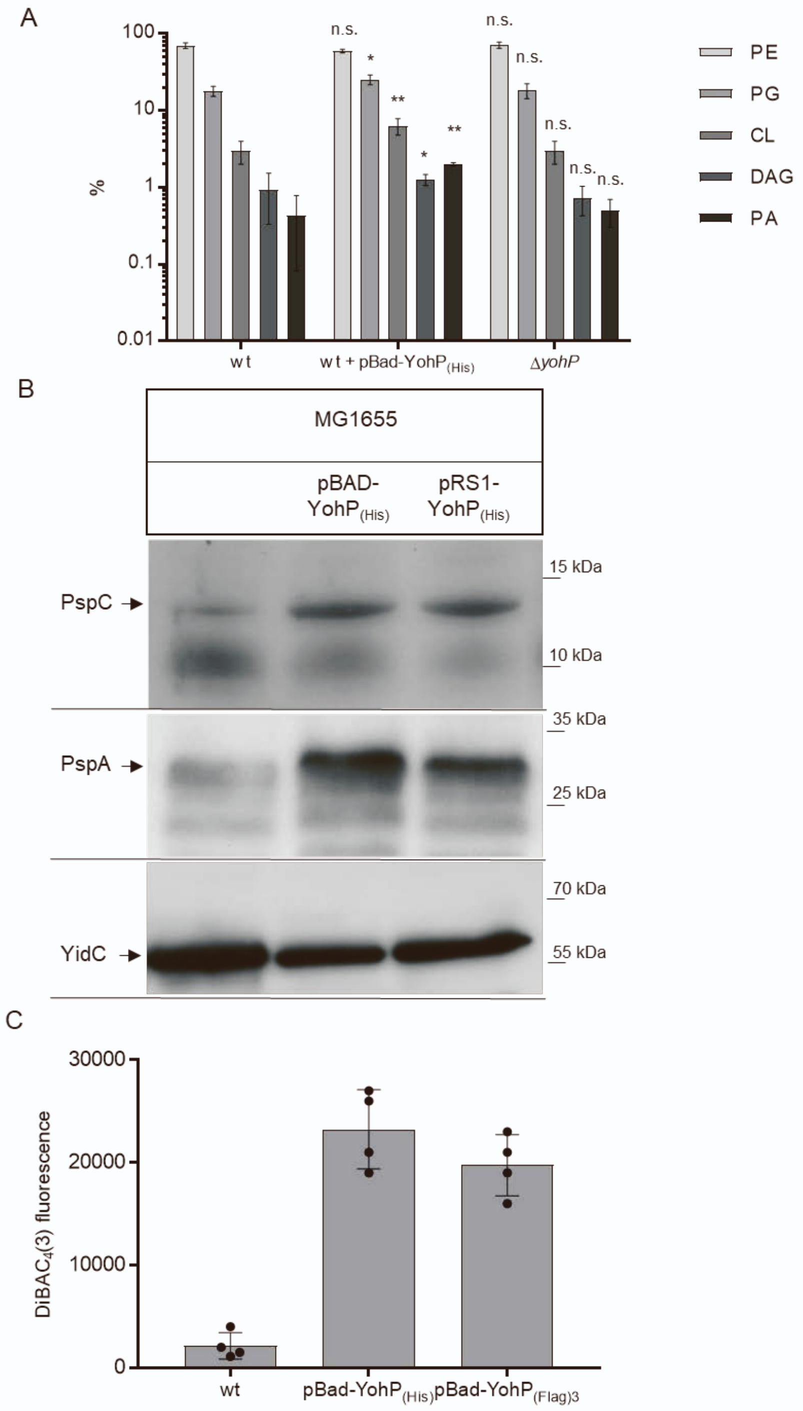

Fig. S7: Premature YohP production is linked to changes in the lipid composition and induces the phage-shock response (Related to Figs. 5-7). **A.** Lipidomics analyses were performed and analyzed as described in the legend to Fig. 5B. The data of Fig. 5 are complemented here by data for diacylglycerol (DAG) and phosphatidic acid (PA). **B**. The indicated E. coli cells were grown on LB medium up to an optical density of 1.5. Expression of *yohP* from the plasmids was induced at OD_{600} = 0.4 with 1 mM IPTG or 0.2% arabinose. Cells were then processed as described in the legend to Fig. S2 and analyzed with antibodies against PspC, PspA, and YidC. **C.** The membrane potential of the indicated strains was determined as described in Fig. 6A.

Table S1: Differentially expressed proteins in the Δ*yohP* strain (Table S1.exe).

Table S2: Differentially expressed proteins in the *yohP*-expressing strain (Table S2.exe).

Table S3. Oligonucleotide primers used in this study

Name	purpose	sequence (5´-3`)
pRS1-fw	pRS1 opening for Flag-tag cloning	taactcgagtagcataaccccttg
pRS1-rev	pRS1 opening for Flag-tag cloning	aaatatcatcttaaatacgccagtcacc
pRS1-Flagx3	3x Flag sequence attachment to yohP	gcgtatttaagatgatatttggcggcggcagcgactacaaggaccacgacgg-cgactacaaggaccacgacatcgactacaaggacgacgacgacaagtaact cgagtagcataaccc
pBad24-fw	pBad24 opening for Flag-tag cloning	taaatggtacccggggatcctctag
pBad24-rev	pBad24 opening for Flag-tag cloning	aaatatcatcttaaatacgccagtcacc
pBAd24-Flag3	3x Flag sequence attachment to yohP	gcgtatttaagatgatatttggcggcggcagcgactacaaggaccacgacgg-cgactacaaggaccacgacatcgactacaaggacgacgacgacaagtaaat ggtacccggggatcc
CAT-fw	Cm^R cartridge flanked by FRT sites	gtgtaggctggagctgcttc
CAT-rev	Cm^R cartridge flanked by FRT sites	atgggaattagccatggtcc
YohP-Cm-fw	*yohP* indel	ggcttcgttttttcctatacttattcagcactcacaaataaaggaacgcc-agtgtaggctggagctgcttc
YohP-Cm-rev	*yohP* indel	ggaccatggctaattcccataattaattaatgtcatcaggtccgaaaata-acgagaatatttcagtctctc
YohP-control fw	control Δ*yohP* strain	cctatacttattcagcactcac
YohP-control rev	control Δ*yohP* strain	ctcgttattttcggacctgatgac

:
