# Supplementary material for: Metabolic silencing induced by the small bacterial membrane protein YohP
Source: iScience. 2025 Nov 19;28(12):114123. doi: 10.1016/j.isci.2025.114123 (PMC12719787; doi:10.1016/j.isci.2025.114123)
Supplement: Data S1. Differentially expressed proteins in the ΔyohP strain [file mmc4.zip › Data sets_Suppl. material/Supplementary figures.pptx]

## Slide 1
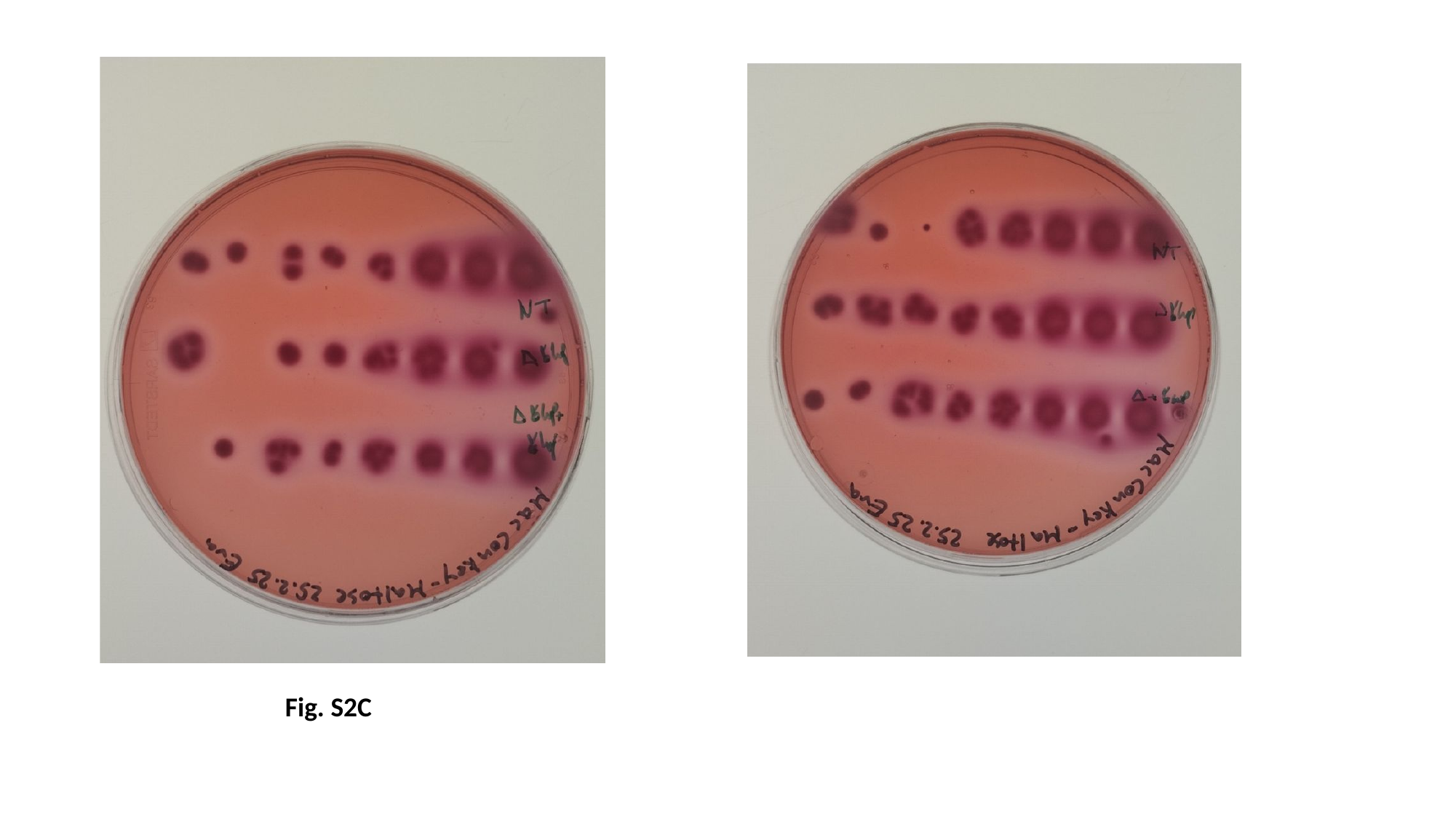

Fig. S2C

## Slide 2
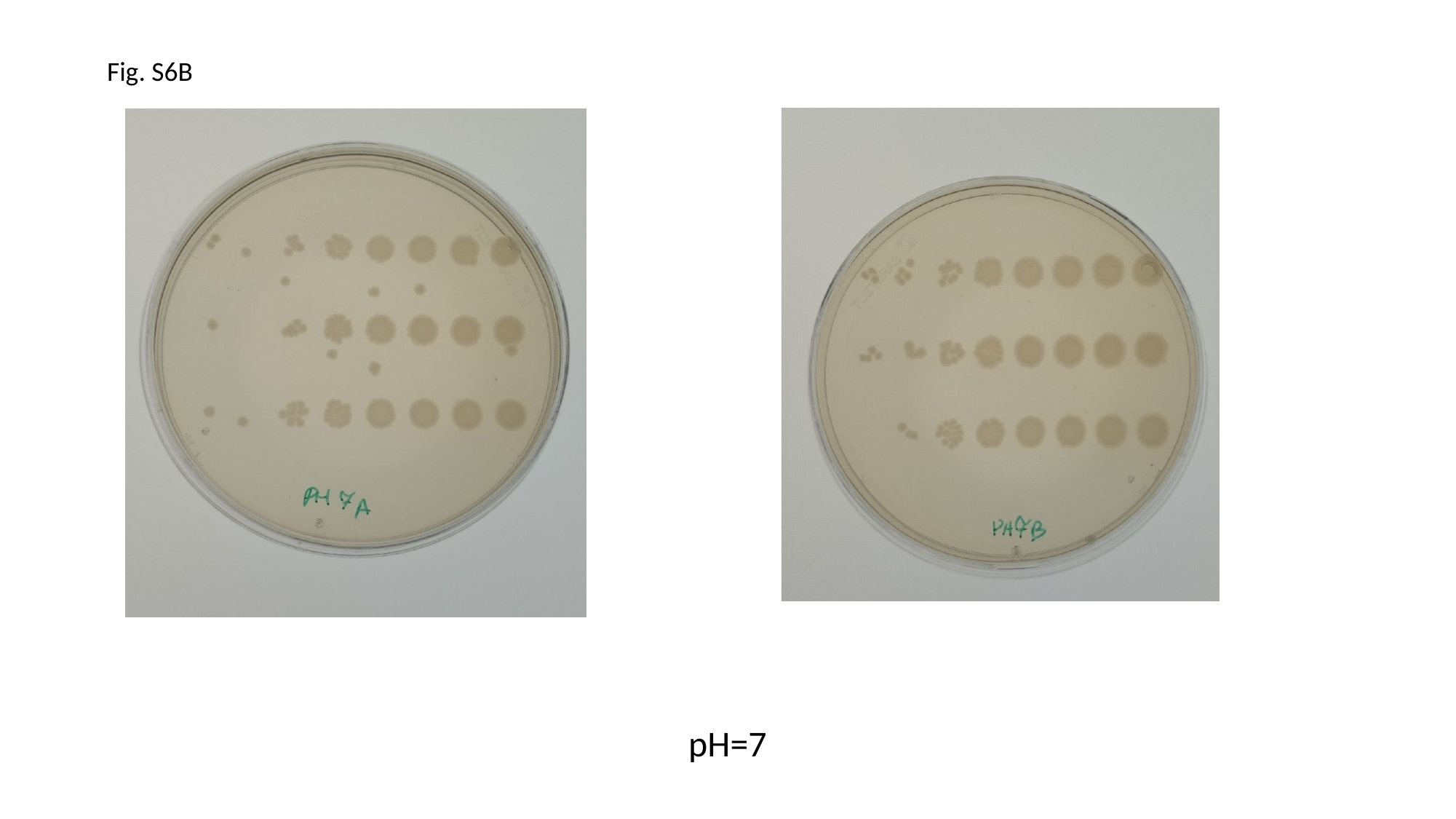

Fig. S6B
pH=7

## Slide 3
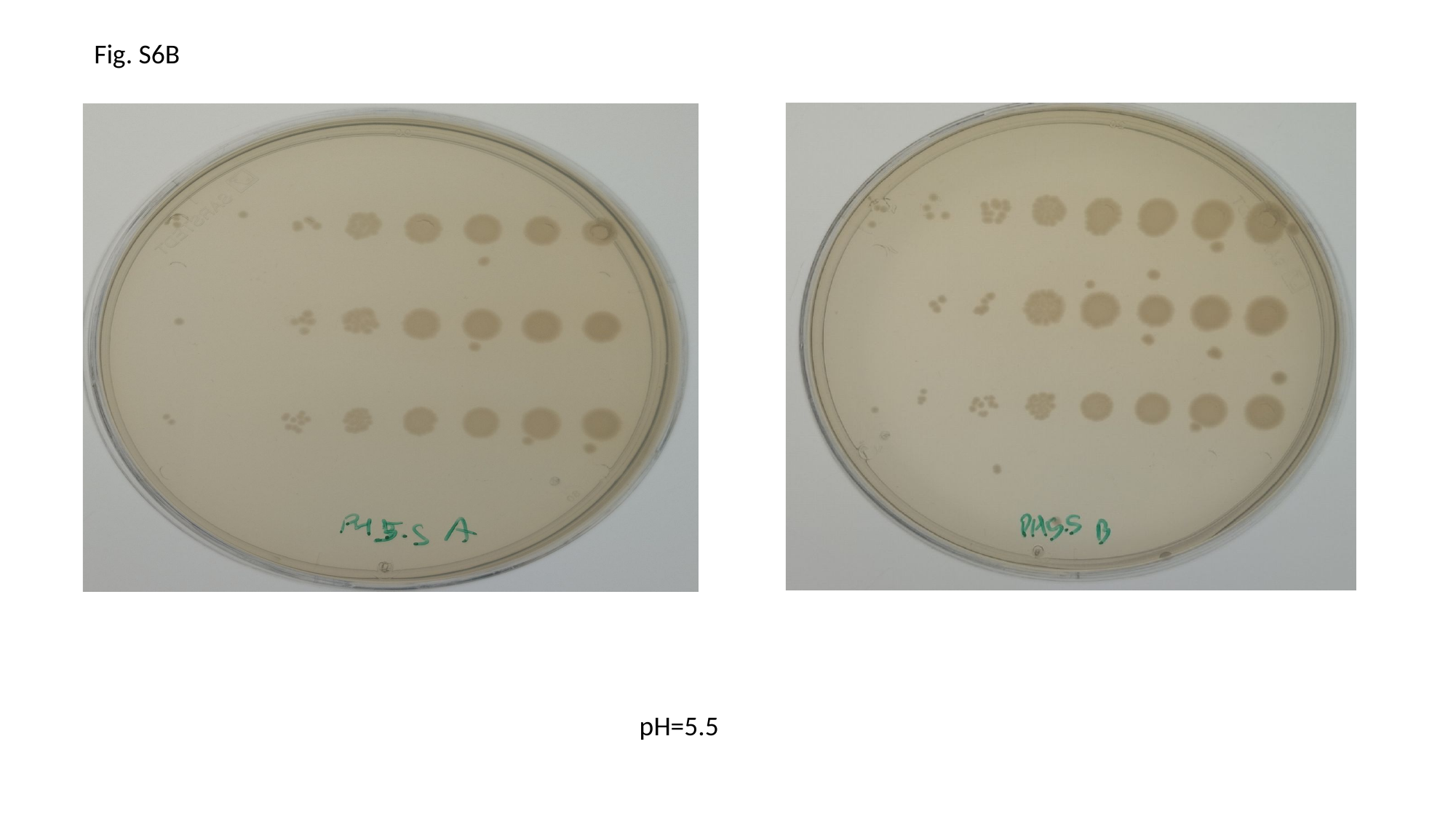

Fig. S6B
pH=5.5

## Slide 4
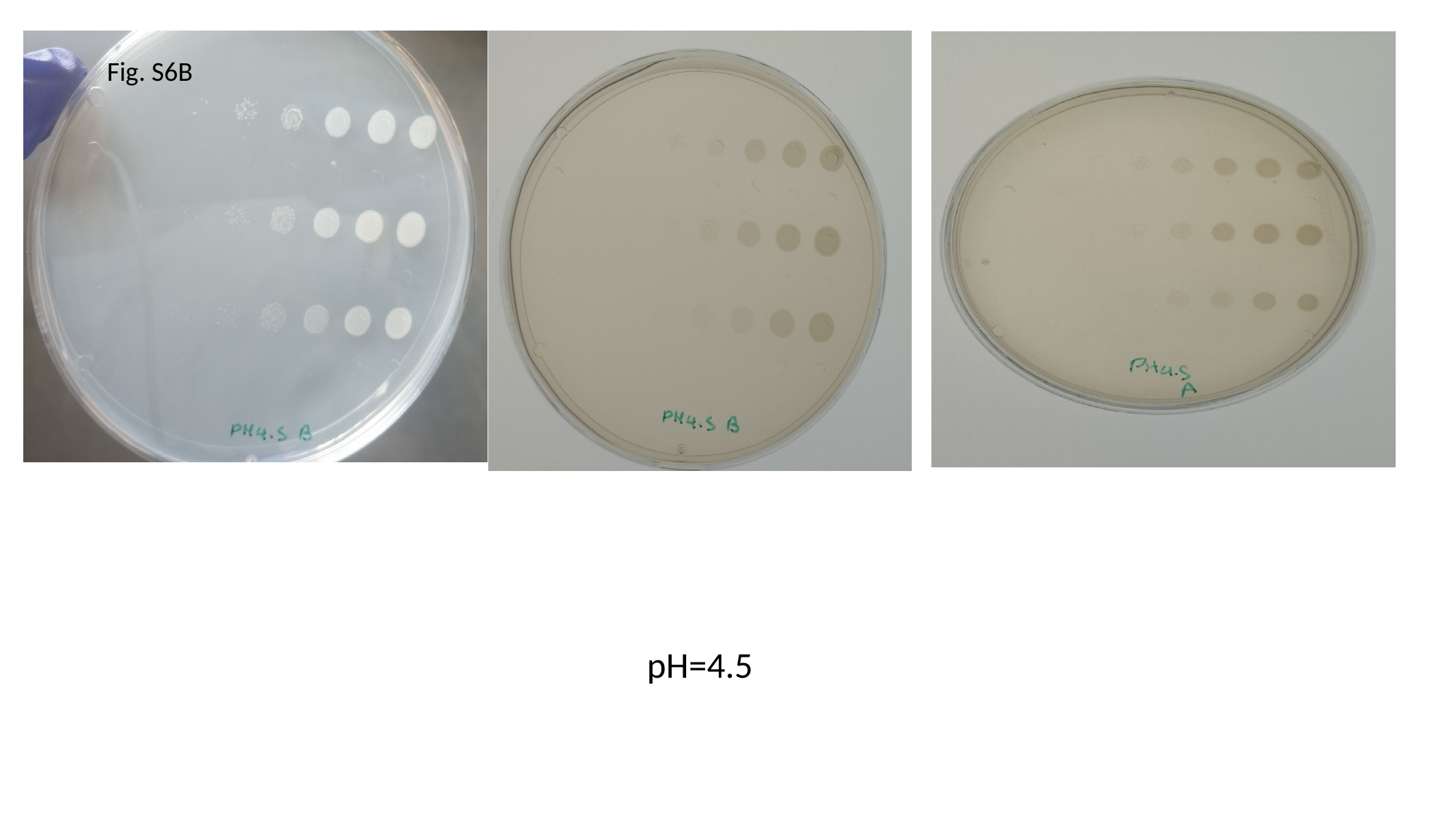

Fig. S6B
pH=4.5

## Slide 5
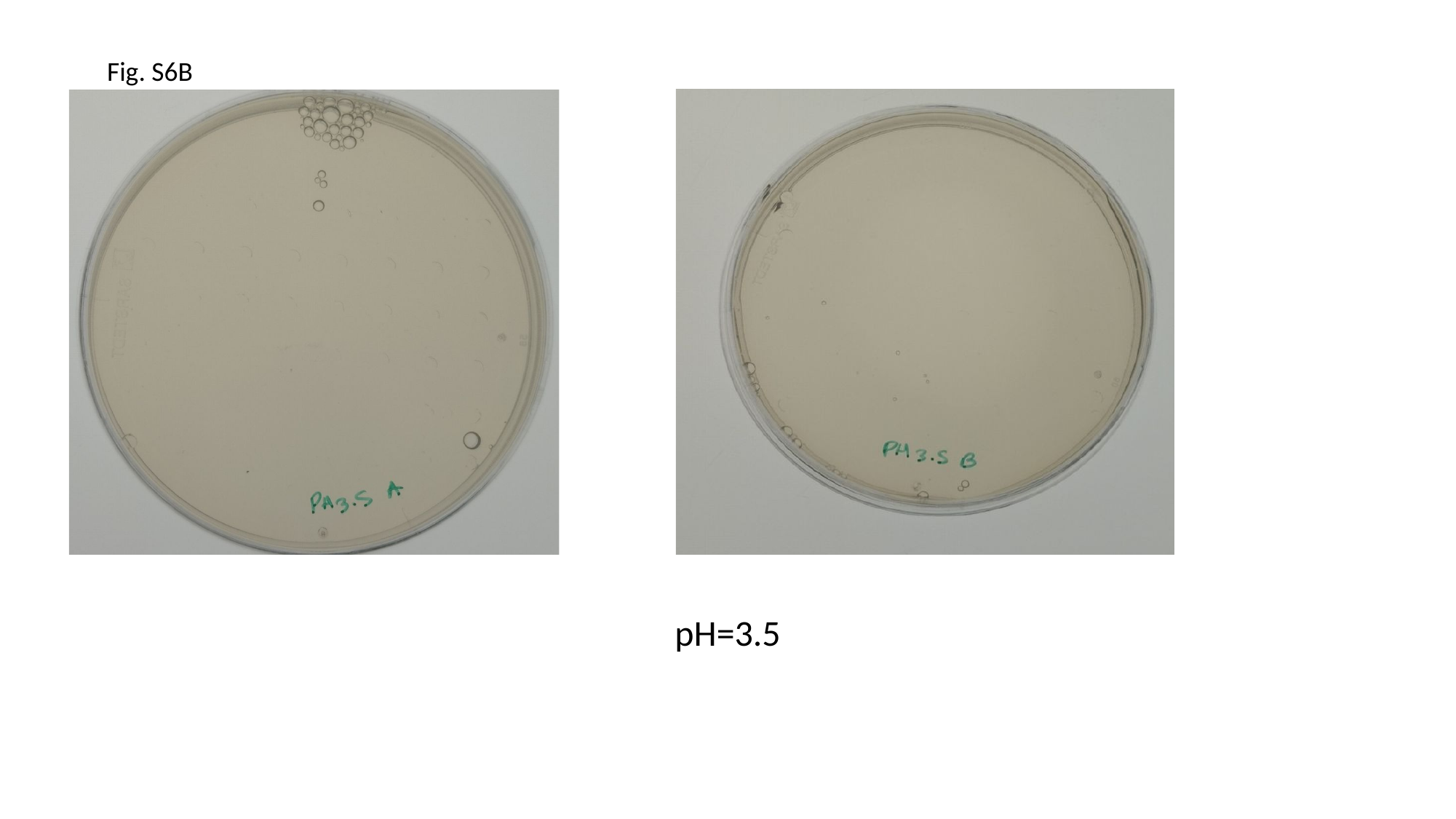

Fig. S6B
pH=3.5

## Slide 6
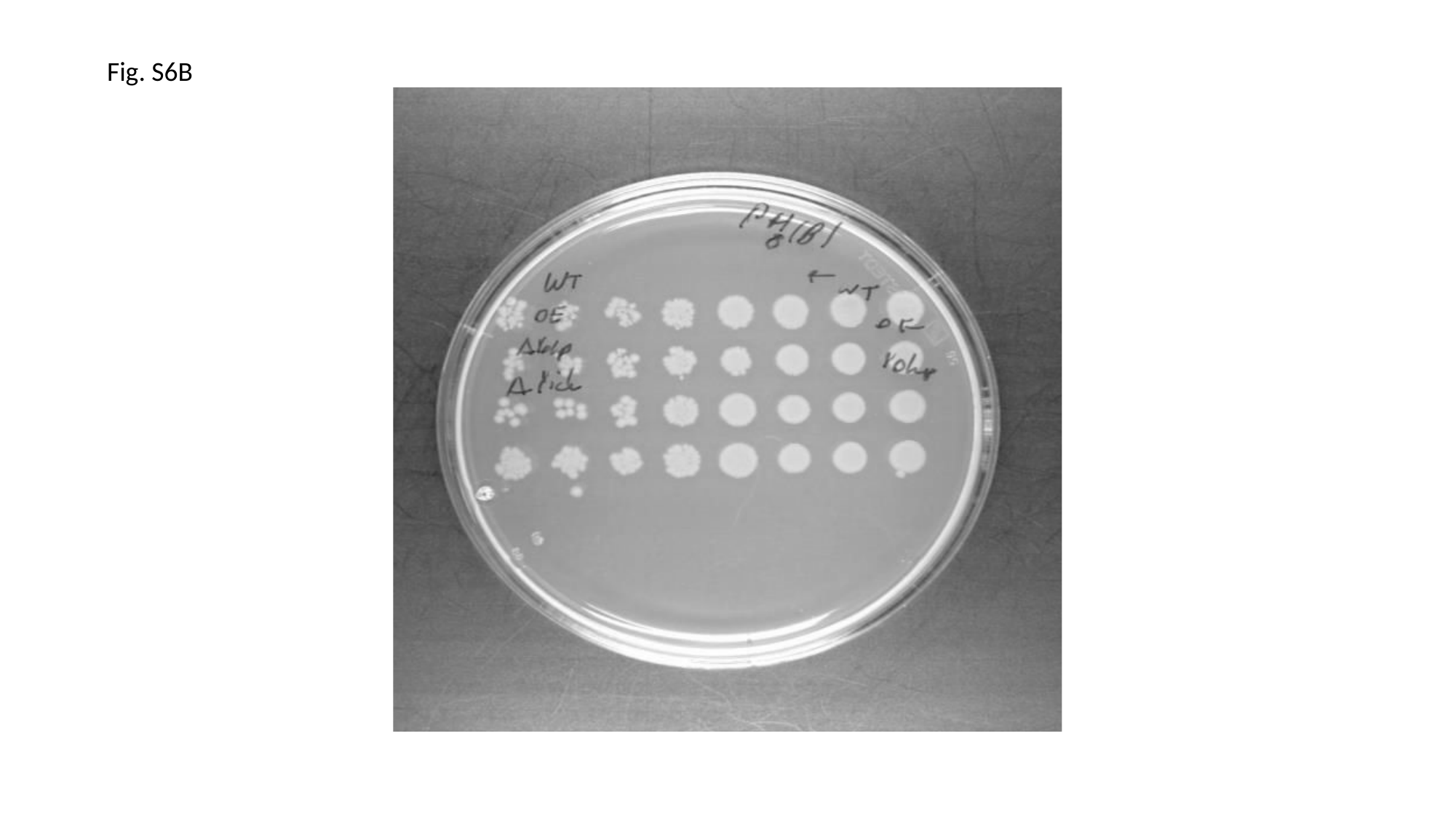

Fig. S6B

## Slide 7
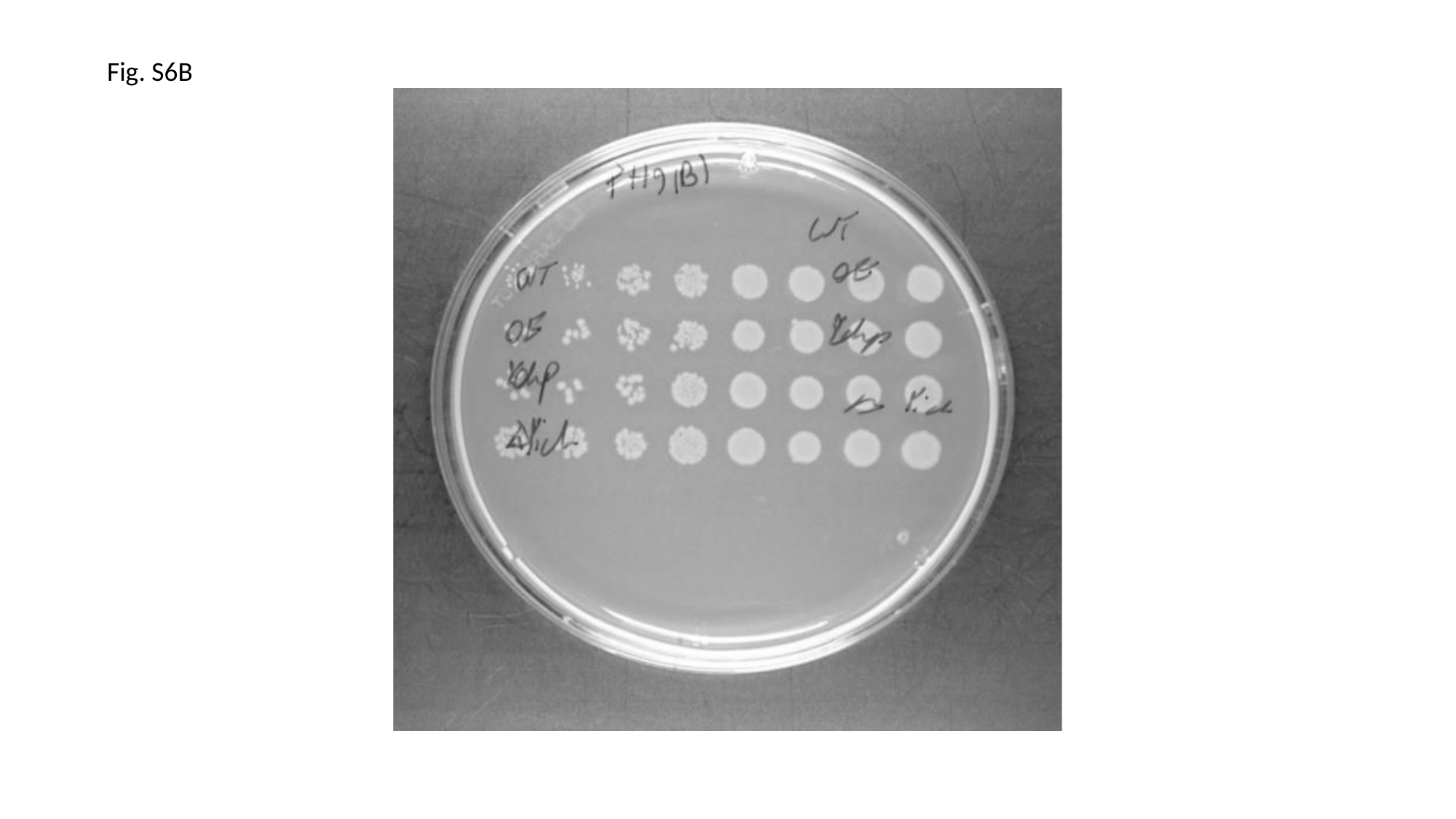

Fig. S6B

## Slide 8
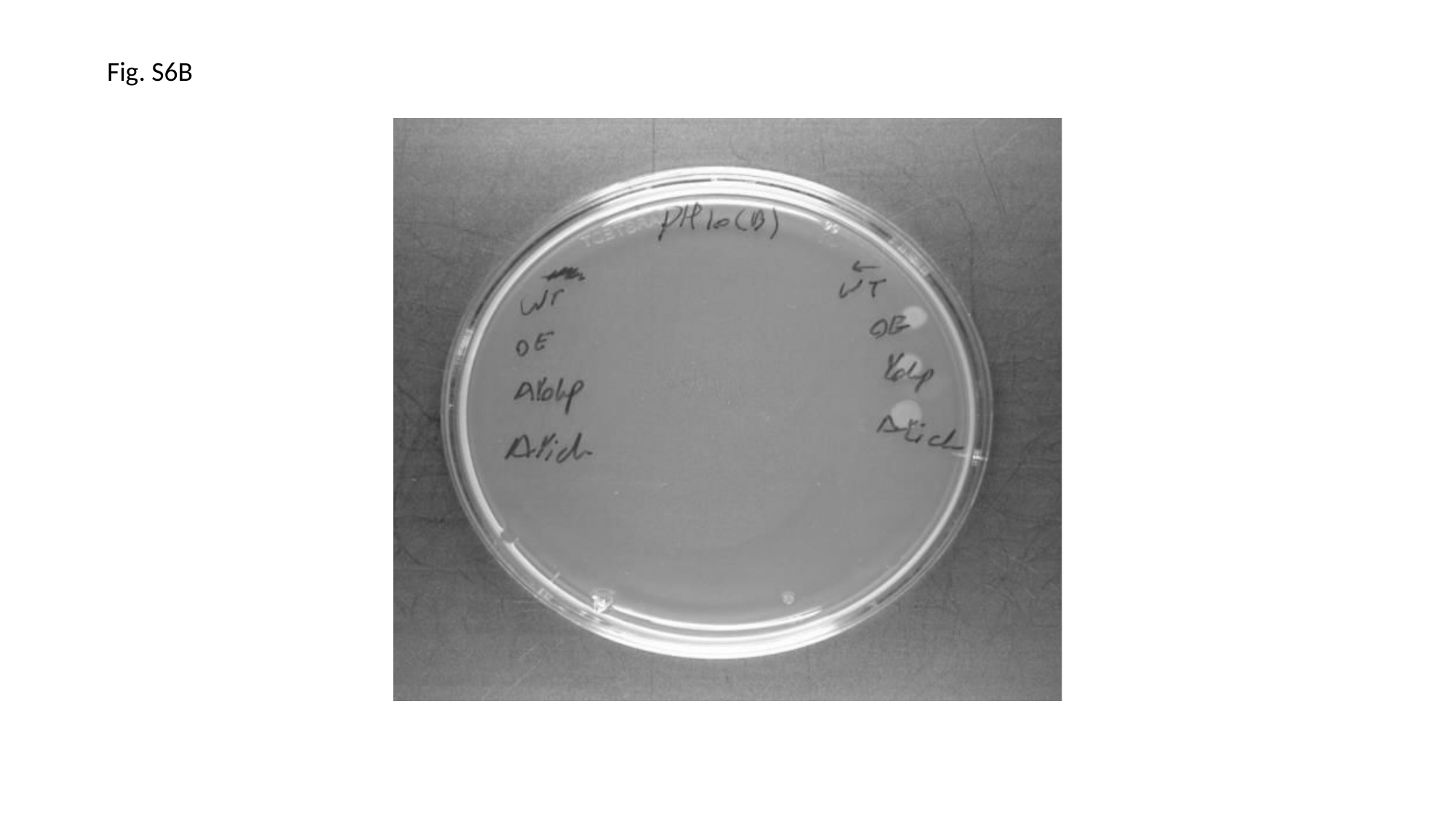

Fig. S6B
