# Supplementary material for: Metabolic silencing induced by the small bacterial membrane protein YohP
Source: iScience. 2025 Nov 19;28(12):114123. doi: 10.1016/j.isci.2025.114123 (PMC12719787; doi:10.1016/j.isci.2025.114123)

Fig. 1A_YohP

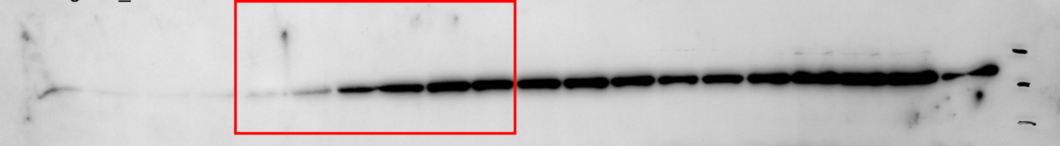

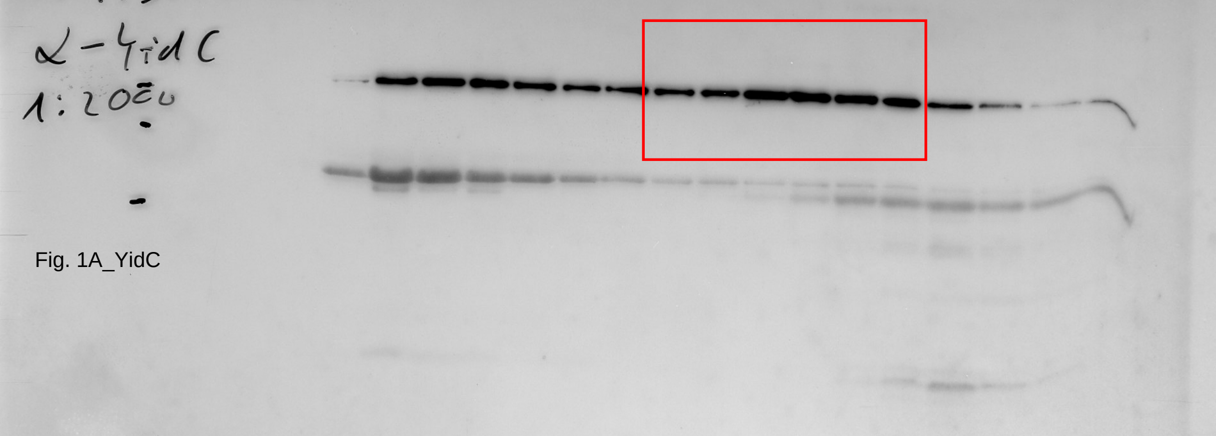

Fig. 1A_YidC

Fig. 1B

Fig. 1D

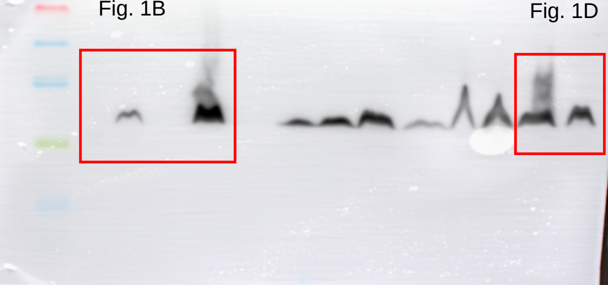

Fig1C

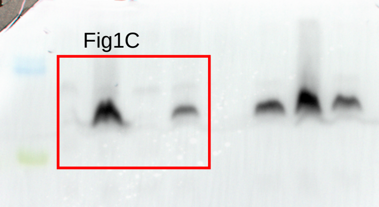

Fig. 1E

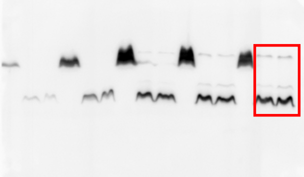

Fig. 2A

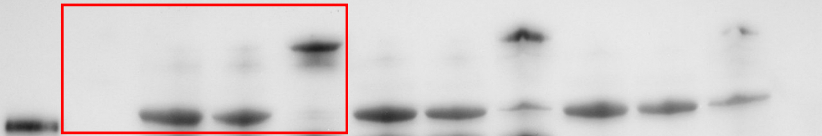

Fig. 2A

YidC

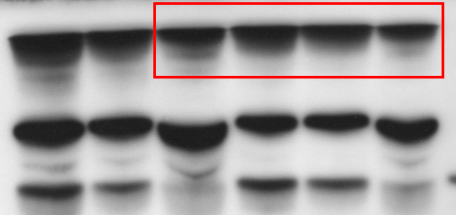

Fig. 2B; YohP

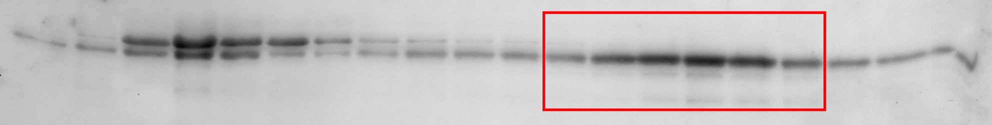

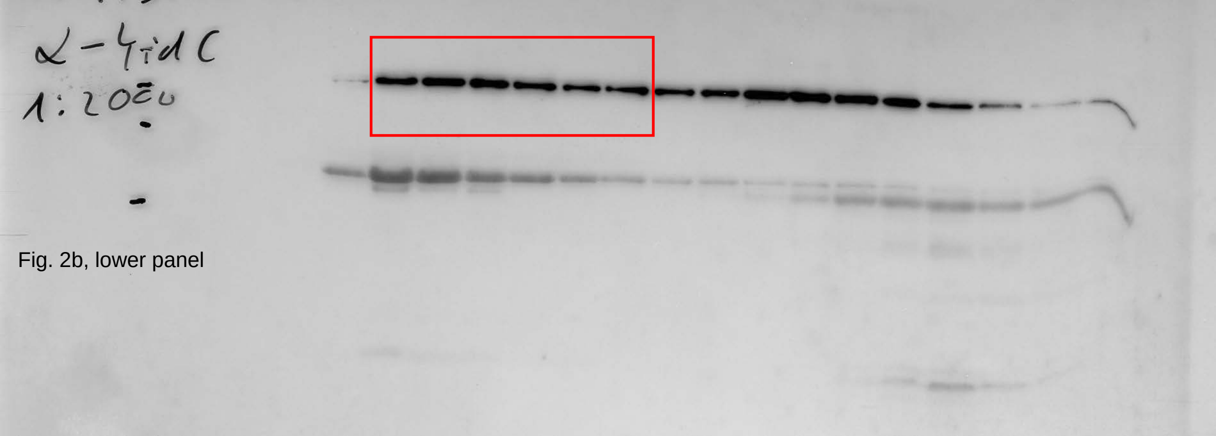

Fig. 2b, lower panel

Fig. 4D; TnaA

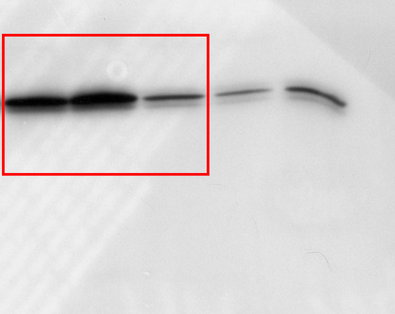

Fig. 4D_YidC

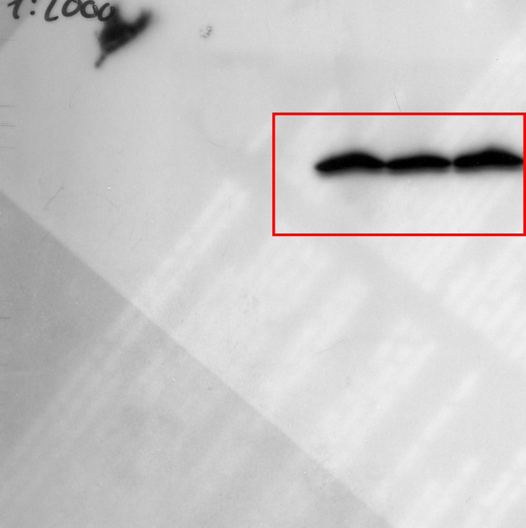

Fig. 4E, left

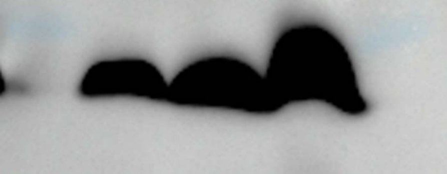

Fig. 4E, right

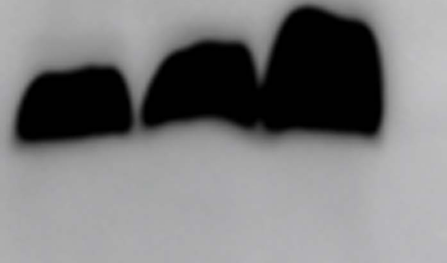

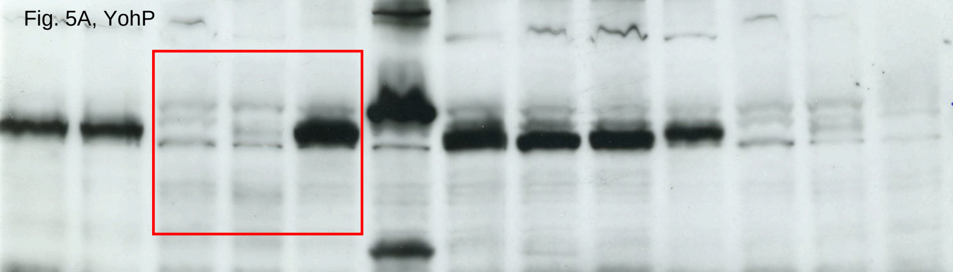
Fig. 5A, YohP

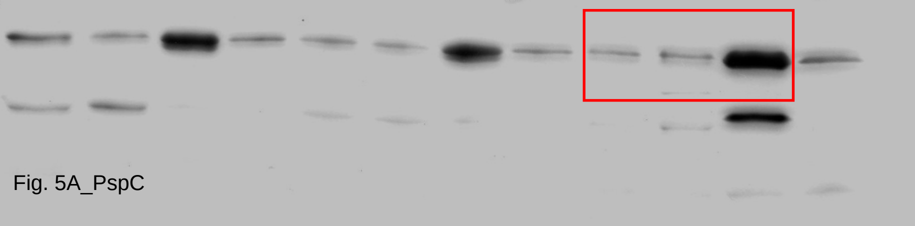

Fig. 5A_PspC

Fig. 5A_PspA

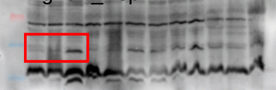

Fig. 5A_RpoS

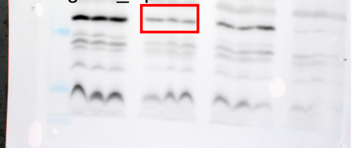

Fig5A_YidC

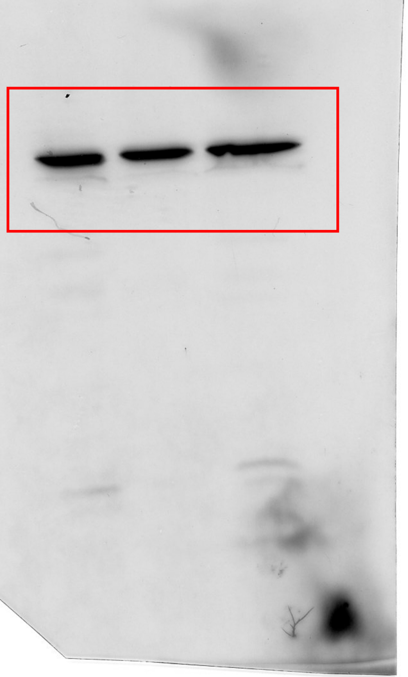

Fig. 7B_ pyrD

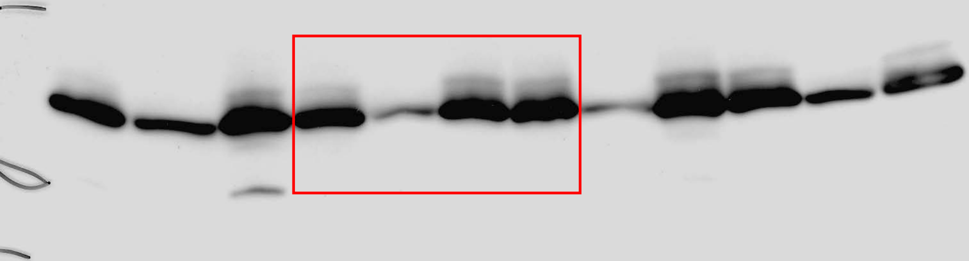

Fig. 7B_YohP

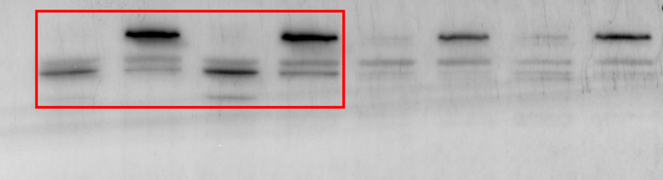

Fig. 7B_YidC

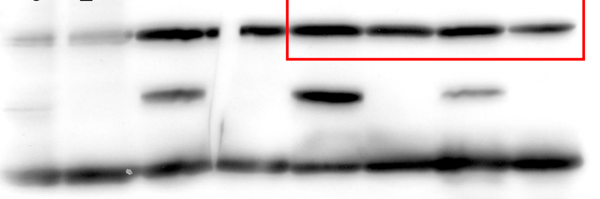

Supplement: Data S2. Differentially expressed proteins in the yohP-expressing strain [file mmc5.zip › Western-blots/Western_Blots Main manuscript.pdf]
