# Supplementary material for: Metabolic silencing induced by the small bacterial membrane protein YohP
Source: iScience. 2025 Nov 19;28(12):114123. doi: 10.1016/j.isci.2025.114123 (PMC12719787; doi:10.1016/j.isci.2025.114123)
Supplement: Data S2. Differentially expressed proteins in the yohP-expressing strain [file mmc5.zip › Western-blots/Western_Blots Suppl. material.pdf]

Fig. S2A, YohP- left

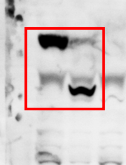

Fig. S2A,
YohP,
right

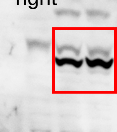

Fig S2A, left & right YidC controls

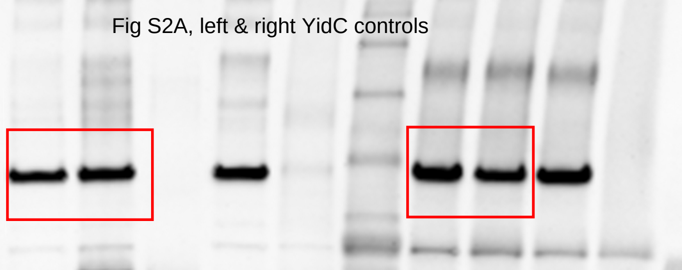

Fig. S6A_TnaA

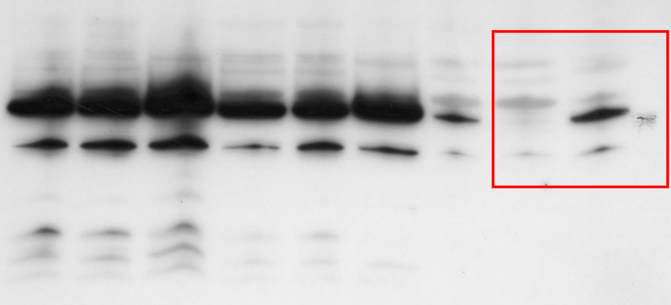

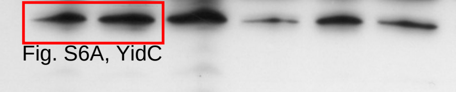

Fig. S6A, YidC

Fig. S7B_PspC

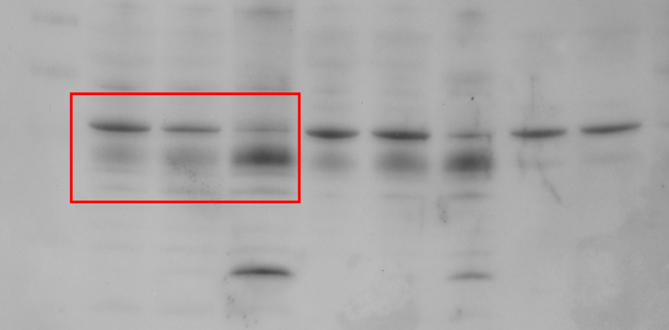

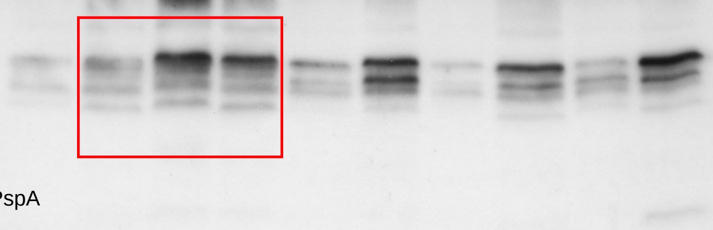

Fig. S7B; PspA

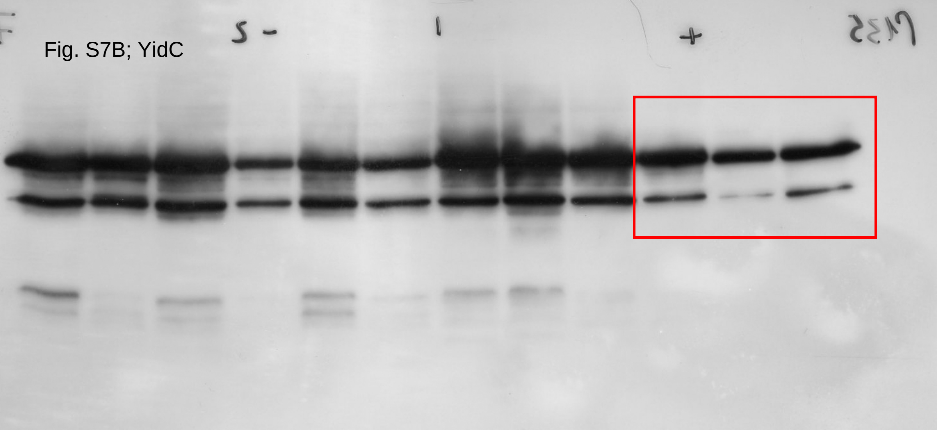

Fig. S7B; YidC
